# Dynamic population coding of kinematic structure across executed and observed actions in primate premotor cortex

**DOI:** 10.1126/sciadv.aed9309

**Published:** 2026-06-19

**Authors:** Konstantinos Chatzimichail, Christos Paschalidis, Eleftheria Tzamali, Vassilis Papadourakis, Vassilis Raos

**Affiliations:** ^1^ Lab of Movement Physiology, Medical School, University of Crete, Heraklion, Greece.; ^2^ Graduate Program in the Brain and Mind Sciences, University of Crete, Heraklion, Greece.; ^3^ Institute of Applied and Computational Mathematics, Foundation for Research and Technology-Hellas, Heraklion, Greece.

## Abstract

Neurons active during both action execution and observation [mirror neurons (MirNs)] are central to theories of action understanding, yet what they represent remains debated: abstract goals, static grips, or movement kinematics? We recorded 433 neurons from macaque premotor cortex during execution and observation of reach-to-grasp actions. Population analyses revealed grasp-specific information in both conditions, broadly distributed across neurons and dynamically reconfigured over time. Generalization across task phases was limited, indicating time-specific and evolving population codes rather than static representations. Execution and observation were linked by a shared, partially overlapping population geometry that supported reliable cross-condition classification, with the strongest alignment emerging during movement and hold. Neural activity was systematically related to multidimensional hand kinematics, and these relationships generalized across neuronal populations and across agents. Together, these findings support a dynamic population-level account of MirN function in which premotor circuits integrate visual and motor signals to represent and anticipate the unfolding structure of others’ actions.

## Teaser

Premotor neural populations dynamically integrate seeing and doing by reflecting the unfolding kinematics of grasping actions.

## INTRODUCTION

How does the brain transform the sight of another’s movement into an internal motor representation? This question lies at the core of social interaction and has long driven the study of mirror neurons (MirNs). First identified in the ventral premotor cortex of macaques, these visuomotor neurons discharge both when an animal executes an action and when it observes the same action performed by others (*1*, *2*). This dual response property led to the proposal that MirNs provide a neural substrate for understanding others’ actions (*3*). Yet, three decades later, the representational properties and functional role of MirNs remain actively debated (*4*–*8*).

A dominant hypothesis holds that MirNs encode the goal of an action, meaning the intended outcome toward which the action is directed, rather than its motor details (*9*–*11*). Consistent with goal-selective coding, neurons often respond differently when the same object is grasped for different purposes (e.g., grasping-to-eat versus grasping-to-place) (*12*, *13*). However, other studies report that MirNs are selective for grip type even when the goal is constant (*2*, *14*–*17*), challenging a purely goal-based account. At the population level, ensembles robustly encode grip information during action execution but much less consistently during observation (*18*, *19*), suggesting that the relationship between executed and observed representations is more complex than either a purely goal-based or purely motor account would predict.

An alternative, nonexclusive hypothesis is that MirNs reflect the kinematic structure of actions (*15*). This perspective was central to the original “direct matching” framework but was largely dismissed after early reports suggested that MirNs fail to respond to intransitive movements (*2*, *20*), which was interpreted as evidence for goal-based rather than kinematic coding (*21*, *22*). Subsequent studies, however, demonstrated that most MirNs do respond to intransitive movements, albeit less vigorously than to transitive ones (*15*, *16*, *23*). Moreover, psychophysical research has shown that actions directed at the same object but serving different goals often display distinct kinematic profiles (*24*–*27*), raising the possibility that some goal-selective responses may instead reflect sensitivity to movement dynamics.

By definition, MirNs should exhibit similar coding during execution and observation. Yet, since their initial discovery, only a minority have shown strict congruency, even under lenient criteria such as responding most strongly to the same grip across both conditions (*2*). Using quantitative indices, we previously reported that single-neuron congruency in both dorsal and ventral premotor cortex is at chance levels (*15*), a finding confirmed by more recent studies (*28*). This has led to the hypothesis that execution-observation matching may emerge at the population level rather than within individual neurons (*16*, *29*). However, whether executed and observed actions share a common population-level representational format, partially overlapping subspaces, or distinct but systematically related codes remains unresolved.

Consequently, we posed three key questions about MirNs: (i) How is action information represented in MirN ensembles? (ii) Do observed and executed actions share a common population-level code? (iii) How does MirN activity relate to the kinematics of observed and executed actions? Because MirNs represent only a subset of grasp-related neurons in premotor cortex and constitute a minority of neurons active during grasp execution, focusing exclusively on this population would provide only a partial view of premotor representations. A comprehensive account therefore requires considering neurons that are active during action execution but show no modulation during action observation (non-MirNs). Their inclusion allows us to address a fourth question: Are the representational properties observed in MirNs unique to neurons exhibiting mirror responses, or are they shared more broadly across premotor neurons involved in grasp execution?

To address these questions, we recorded a spiking activity from 433 neurons in dorsal (PMd) and ventral (PMv) premotor cortex of macaques performing and observing reach-to-grasp actions. Using population decoding, regression modeling, cross-temporal and cross-conditional analyses, and population-subspace approaches, we show that grip information is dynamically distributed across premotor populations, that execution and observation share a partially aligned representational format within the MirN population, and that premotor activity closely reflects the evolving kinematic structure of actions. Together, these findings reveal a population-level mechanism by which premotor ensembles may transform observed actions into predictive motor representations.

## RESULTS

A total of 433 neurons were recorded from macaque monkeys while they either executed reach-to-grasp actions or observed an experimenter performing reach-to-grasp actions directed toward a set of objects designed to elicit different grasp configurations. Of these, 285 neurons were active during both execution and observation and were therefore classified as MirNs, whereas 148 neurons were active exclusively during action execution and showed no modulation during observation and were classified as non-MirNs. Among these, 240 MirNs and 129 non-MirNs were recorded in at least nine trials per grip and condition and comprised the datasets analyzed in this study. A detailed account of the recorded neurons per area and animal is provided in table S1.

**Fig. 1.**
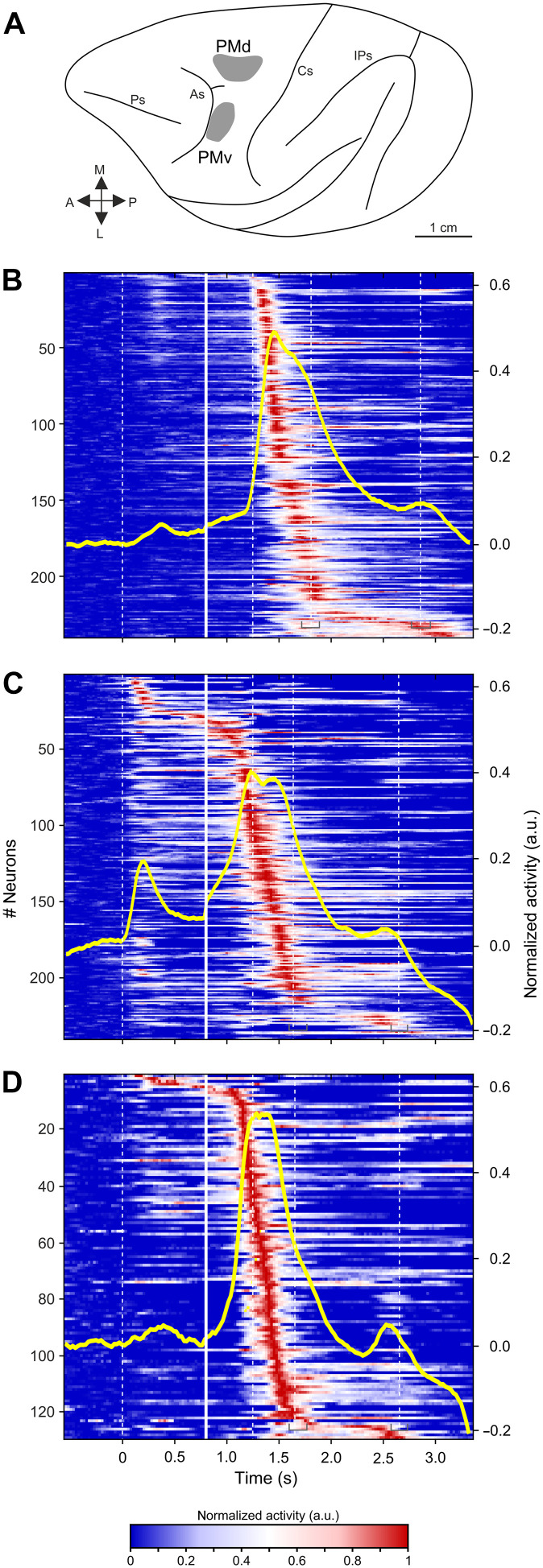
Neurons recorded in the premotor cortex. MirNs were active during both action execution and action observation, whereas non-MirNs were active only during action execution. (**A**) Schematic of the left hemisphere of the monkey brain showing the recording sites in PMd and PMv. Shaded areas represent the extent of recoded regions across both monkeys. As, arcuate sulcus; Cs, central sulcus; IPs, intraparietal sulcus; Ps, principal sulcus; A, anterior; L, lateral; M, medial; P, posterior. (**B** to **D**) Time course of normalized MirN activity during grasp observation (B), grasp execution (C), and non-MirN activity (D). Neural activity is aligned to object presentation onset (first dashed line, time = 0 s) and movement onset (second dashed line, time = 1.25 s). Each row of the heatmaps represents a single neuron, sorted independently within each panel by the time of peak activity in that condition. The color scale indicates normalized firing rate. Thick yellow traces show the population mean across neurons (normalized firing rate). The third and fourth dashed vertical lines correspond to the median end of movement and end of the hold phase, respectively; brackets indicate the 25th to 75th percentiles of these behavioral events. Thick white vertical lines indicate alignment discontinuities introduced by the double alignment. a.u., arbitrary units.

Because the findings were replicated in both cortical areas, data from PMd and PMv were pooled for subsequent analyses. Recording sites are shown in Fig. 1 and fig. S1. An outline of the behavioral tasks is provided in fig. S1 together with images of the objects and schematic representations of the instructed grasp configurations. The time course of neural activity across tasks is illustrated in Fig. 1. Some neurons in this dataset were previously described in detail (*15*, *16*).

### Neural population encoding of grasp configurations

**Fig. 2.**
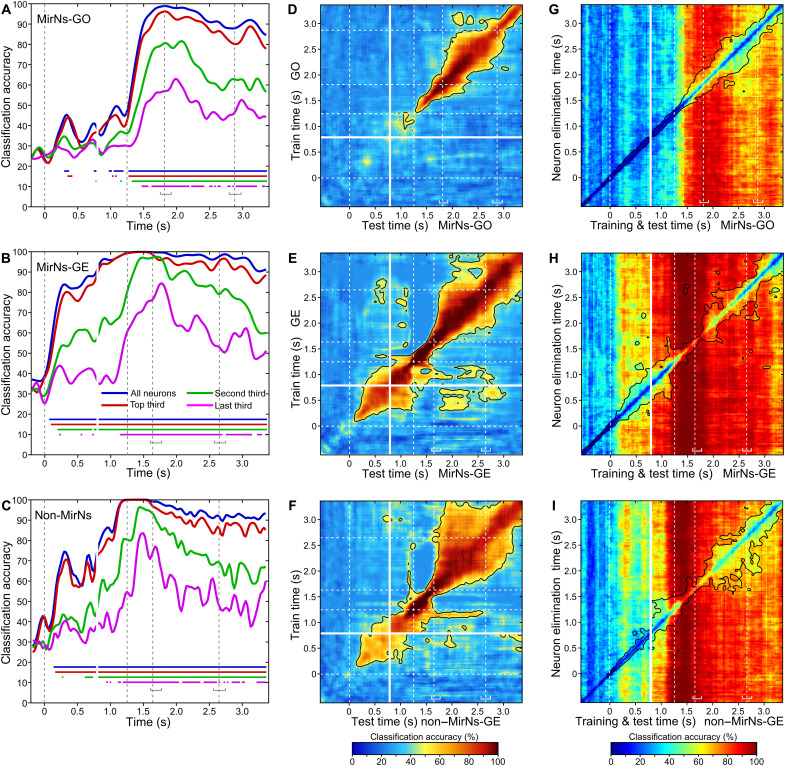
Grasp configurations are classified through a distributed and dynamic neural population code. Decoding was performed on pseudopopulations constructed across recording sessions using a Poisson naïve Bayes classifier. (**A** to **C**) Time-resolved classification accuracy for grasp configuration using all neurons (blue) or subsets with different degrees of selectivity. For MirNs [(A): GO; (B): GE], classification used the 80 most selective neurons at each time bin (red), neurons ranked 81 to 160 (green), or the 80 remaining neurons after excluding the top 160 (violet). For non-MirNs (C), classification used the 43 most selective neurons (red), neurons ranked 44 to 86 (green), or neurons excluding the top 86 at each time bin (violet). Horizontal lines indicate time periods at which accuracy exceeded the permutation-derived null distribution (nonparametric permutation test; 500 label shuffles; one-sided *P* < 0.002). Dashed vertical lines at 0 and 1.25 s mark object presentation and movement onset; later dashed lines indicate median movement and hold-phase endpoints; brackets below show the 25th to 75th percentiles of these behavioral events. (**D** to **F**) Cross-temporal classification matrices for MirNs [(D): GO; (E): GE] and non-MirNs (F). Classifiers were trained at each time bin and tested across all bins. Color scale indicates classification accuracy. Black contours mark significant classification (nonparametric permutation test; 500 label shuffles; one-sided *P* < 0.002). Contours were smoothed for visualization only. Thick white lines denote alignment discontinuities. (**G** to **I**) Effect of removing the most selective neurons (120 MirNs; 65 non-MirNs) at specific time points (*y* axis) on classification performance across the trial [(G): GO; (H): GE; (I): non-MirNs]. Black contours mark time periods in which performance was significantly reduced relative to random neuron removal (nonparametric permutation test; 500 permutations; one-sided *P* < 0.01).

We first asked whether the neuronal populations carry information about grasp configuration. Decoding analyses were performed on the four trained grasp configurations associated with the four objects. The classifier operated on task labels and does not formally dissociate object identity from grasp configuration; however, each object was linked to a predefined grasp that was explicitly trained and behaviorally monitored, providing a stable correspondence between labels and executed grasp patterns. Using Bayesian classifiers, population responses allowed reliable discrimination among the four trained grasp configurations in MirNs during action observation (GO) and execution (GE), as well as in non-MirNs (Fig. 2, A to C, blue graphs).

During GO, decoding accuracy gradually increased as the experimenter performed the movement, peaked near movement end, and remained significantly above chance during the hold period. No grasp configuration information was detected during the object presentation phase. During GE, both neuronal populations conveyed significant grasp configuration information beginning in the object presentation phase, peaking during movement execution, and remaining elevated throughout the hold period.

Having established population-level decoding, we next examined how grasp-related information was distributed across neurons. Neurons were ranked by grasp selectivity at each time bin, and decoding was repeated using only the top third of the most selective neurons, the middle third, or the remaining population after excluding the top two-thirds. The top third achieved decoding performance comparable to that of the full MirN population in both GO and GE, as well as in non-MirNs (Fig. 2, A to C, red graphs). The middle third also supported highly significant decoding (Fig. 2, A to C, green graphs). Excluding the two most selective thirds abolished decoding during the object presentation phase in GE for both populations but preserved reduced, yet significant, decoding during the movement and hold periods in both GO and GE (Fig. 2, A to C, violet graphs). These findings indicate that grasp-related information is broadly distributed across the population, with substantial although incomplete redundancy in both MirNs and non-MirNs.

We then asked whether grasp-related representations were stable across the trial or evolved dynamically over time. To address this, we performed cross-temporal decoding by training a classifier at each time window and evaluating performance across the full temporal sequence. The resulting two-dimensional (2D) heatmaps showed maximal decoding accuracy when training and testing times were aligned, with limited generalization across distant time points (Fig. 2, D to F). Generalization was most evident during the object presentation phase in GE and during the hold period in both GE and GO. Overall, these results indicate that grasp-related population states are predominantly time specific, with only partial temporal generalization.

Temporal generalization was lowest during the movement period, consistent with the rapid evolution of neural and hand trajectories as the action unfolds. Early in the movement, multiple future action states remain possible, whereas as the action approaches completion and the final configuration becomes constrained, action states become more stable, resulting in improved generalization during the hold phase.

**Fig. 3.**
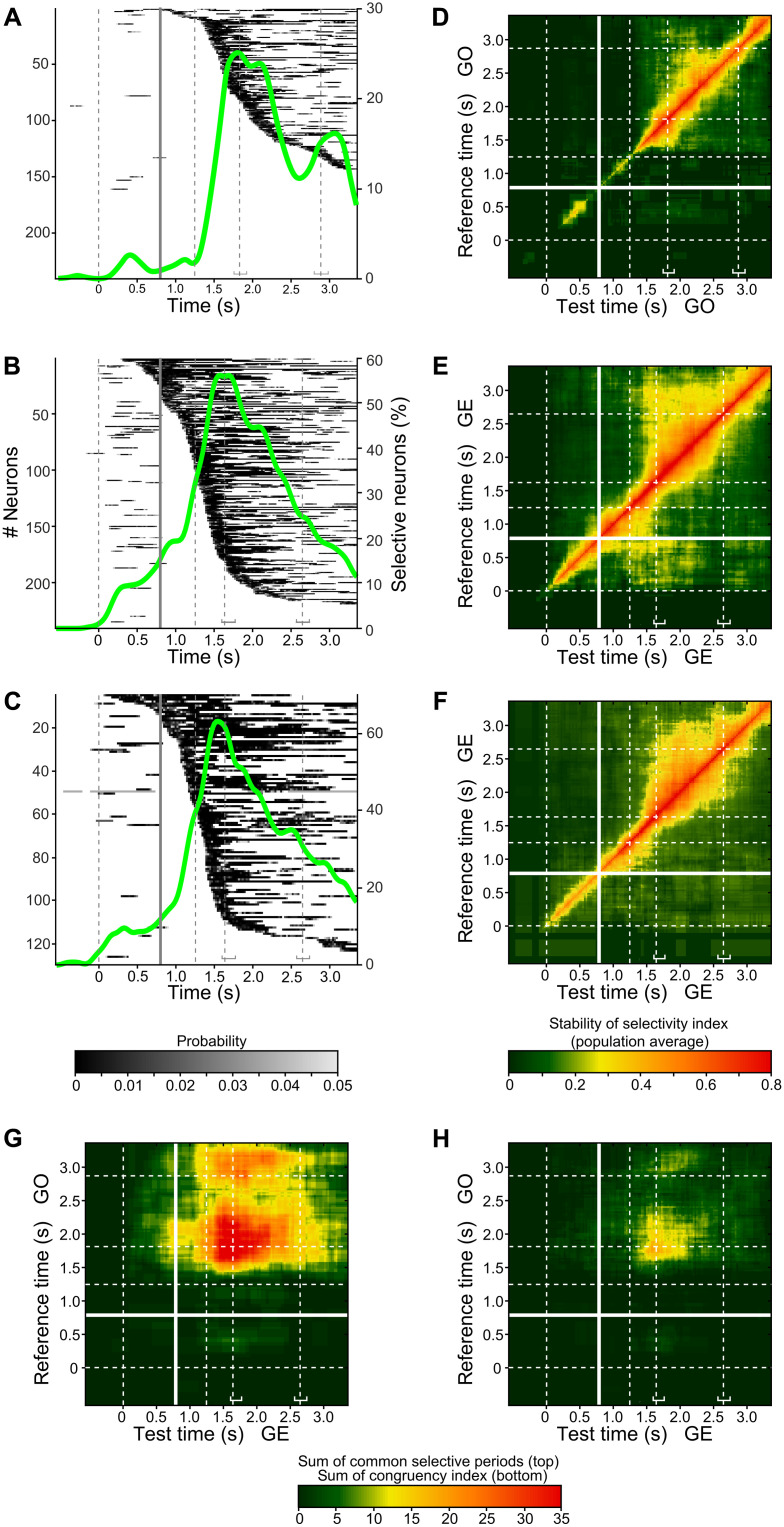
Neuronal selectivity emerges dynamically across time and conditions. (**A** to **C**) Periods of statistically significant grasp selectivity [one-way analysis of variance (ANOVA); factor: four grasp configurations; *P* < 0.05 for ≥7 consecutive bins] for MirNs during GO (A) and GE (B) and non-MirNs (C). Neurons were sorted by the time of their first selective bin, excluding selectivity periods that ended more than 250 ms before movement onset. Grayscale indicates ANOVA *P* values. Thick green trace shows the percentage of selective neurons at each time point. (**D** to **F**) Population selectivity stability. Heatmaps display the normalized sum of positive stability index (SI) values, reflecting persistence of grasp preference across time pairs [(D): MirNs-GO; (E): MirNs-GE; (F): non-MirNs]. Asymmetry arises from normalization relative to the number of selective neurons at the reference time. (**G**) Number of neurons selective in both conditions (GO and GE), across time pairs. (**H**) Population congruency index (CI) across time pairs between GO and GE. Color scales indicate magnitude. Dashed lines mark behavioral events; white lines denote alignment discontinuities.

To investigate the basis of this temporal specificity, we examined how individual neurons contributed grasp-related information across time. The onset and duration of grasp-selective periods revealed that individual neurons became selective at different times and for variable durations (Fig. 3, A to C). We further tested this by removing half of the most selective neurons at each time point from the training set and assessing classifier performance. If the same neurons consistently carried grasp-related information across the trial, then their removal would impair decoding at other time points. Conversely, if different neurons contributed information at different times, then performance reductions would be confined to the time window from which neurons were removed. As shown in Fig. 2 (G to I), removing selective neurons led to decoding impairments restricted to the corresponding and neighboring time windows, consistent with time-specific recruitment of neurons.

We quantified the temporal stability of neuronal selectivity using an index that measured how consistently neurons maintained their grasp preference over time. Heatmaps of this index showed high values primarily along the diagonal, indicating limited persistence of selectivity across extended intervals (Fig. 3, D to F). Notably, temporal generalization increased during the hold period, consistent with stabilization of population states once the final grasp configuration was reached. Together, these findings indicate that grasp-related information is represented by a dynamically reconfigured neuronal ensemble, with time-dependent recruitment and selectivity shifts supporting robust population-level coding.

### Generalization of neural population codes

The dynamic changes in grasp selectivity observed during GO and GE raise the question of whether these dynamics unfold in a comparable manner across conditions. At the single-neuron level, many neurons were selective in both GE and GO, with overlapping periods of selectivity spanning the movement and hold epochs (Fig. 3G). To quantify execution-observation congruency at the level of individual neurons, we computed a congruency index (CI) based on the rank ordering of responses to all grasp configurations across multiple time windows. Unlike previous definitions of congruency which relied on whether the same configuration elicited the strongest response in both conditions or on responses averaged over extended epochs (*2*, *16*), the CI captures the temporal consistency of response structure across conditions using full response rankings. Using this criterion, 67 of 240 neurons displayed a positive CI during the interval from 100 ms before movement onset to the end of the hold phase and were therefore classified as congruent (Fig. 3H).

To determine whether execution and observation share population-level structure beyond single-neuron congruency, we next analyzed population dynamics using principal components analysis (PCA), performed separately for GO and GE. To avoid potential overfitting, we implemented a cross-validated PCA approach in which principal components (PCs) were derived from a subset of trials and variance explained was evaluated on held-out trials as well as on size-matched trials from the opposite condition. The top 10 PCs from each condition accounted for a large proportion of within-condition variance (GO: 67.2%, GE: 65.4%). Cross-projection analyses revealed that GO-derived PCs explained 26.7% of the variance in the execution dataset, whereas GE-derived PCs explained 32.5% of the variance in the observation dataset, indicating partial overlap between execution and observation population subspaces (fig. S2). This overlap was quantified using a normalized alignment index, which was significantly greater than chance (*30*). These results provide an initial assessment of shared population geometry but do not by themselves establish whether overlapping dimensions encode the same task-relevant variables.

**Fig. 4.**
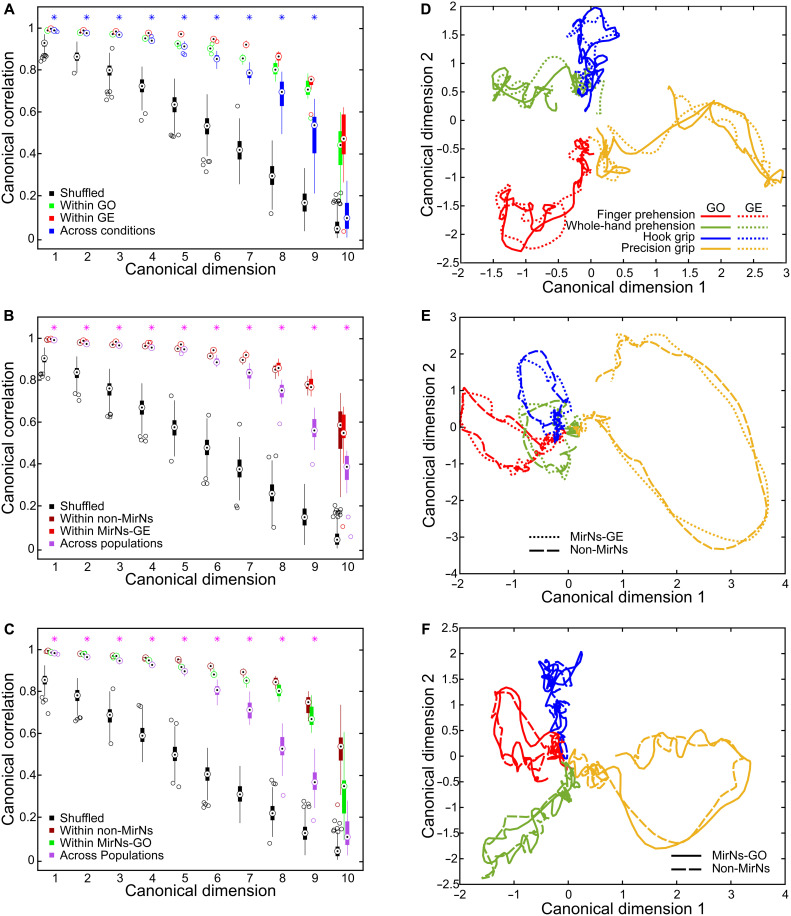
CCA identifies shared structure across conditions and neuronal populations. (**A** to **C**) Box plots show cross-validated canonical correlations across the first 10 canonical dimensions within and between groups. Boxes indicate medians (central dotted circles) and interquartile range (25th to 75th percentiles), whiskers denote non-outlier minima and maxima, and circles indicate outliers. Statistical significance was assessed using nonparametric permutation testing (500 label shuffles; one-sided *P* < 0.01). Black denotes the null distribution derived from shuffled data. (**D** to **F**) Neural activity projected onto the first two canonical dimensions. [(A) and (D)] MirNs (execution and observation). [(B) and (E)] MirN-GE and non-MirN populations. [(C) and (F)] MirN-GO and non-MirN populations. Points represent trial-averaged trajectories for each grasp configuration.

To directly test whether execution and observation population spaces contain aligned grasp-related dimensions, we next applied canonical correlation analysis (CCA). CCA identified pairs of dimensions, one from GE and one from GO, whose grasp-related activity covaried maximally across conditions (Fig. 4A). These results indicate that execution and observation population spaces contain matching representations of grasp-related information. Trajectories of the different grasp configurations in the space defined by the first two canonical dimensions were differentiated, while trajectories corresponding to the same configuration in GE and GO occupied closely related regions, consistent with similar population-level representations across conditions (Fig. 4D). The largely separated grasp configuration trajectories, without abrupt rearrangement or entanglement during the trial, suggest that their relative organization is broadly preserved within the shared execution-observation structure. This pattern is consistent with the view that reduced temporal generalization is unlikely to arise solely from internal rearrangement of grasp representations within a fully stable subspace.

**Fig. 5.**
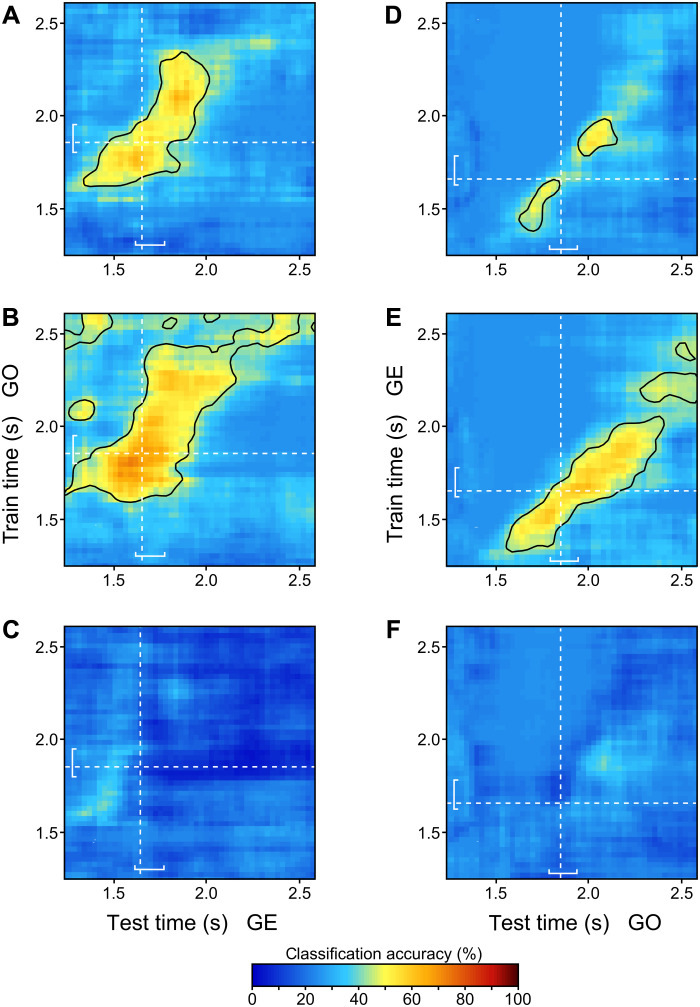
Classification of grasp configuration generalizes across conditions in the original neural space. Cross-temporal classification performed in the original neural space. (**A** to **C**) GO → GE. (**D** to **F**) GE → GO. Classification used all neurons [(A) and (D)], 67 congruent neurons [(B) and (E)], or 67 randomly selected incongruent neurons [(C) and (F)]. Black contours mark significant classification (nonparametric permutation test; 500 label shuffles; one-sided *P* < 0.002). Time aligned to movement onset (1.25 s); dashed lines mark movement end.

We next assessed cross-condition generalization directly in the original population space without dimensionality reduction. In this space, cross-conditional decoding was significant from mid-movement through the hold phase in both directions (Fig. 5, A and D). However, performance was driven exclusively by neurons classified as congruent, consistent with the definition of congruency (Fig. 5, B and E). Because the CI was defined independently of any cross-condition decoding analysis, decoding performance provides an independent test that neurons classified as congruent based on response structure preferentially support execution-observation generalization. Incongruent neurons alone did not yield significant decoding in the original space (Fig. 5, C and F).

Restricting activity to jointly defined dimensions enhanced cross-condition generalization. Using manifold optimization, we defined a shared subspace that maximized variance across both conditions (*29*). Cross-conditional decoding in this shared subspace exhibited longer periods of significant accuracy and higher performance compared to the original space (fig. S3). This enhancement remained primarily dependent on congruent neurons.

**Fig. 6.**
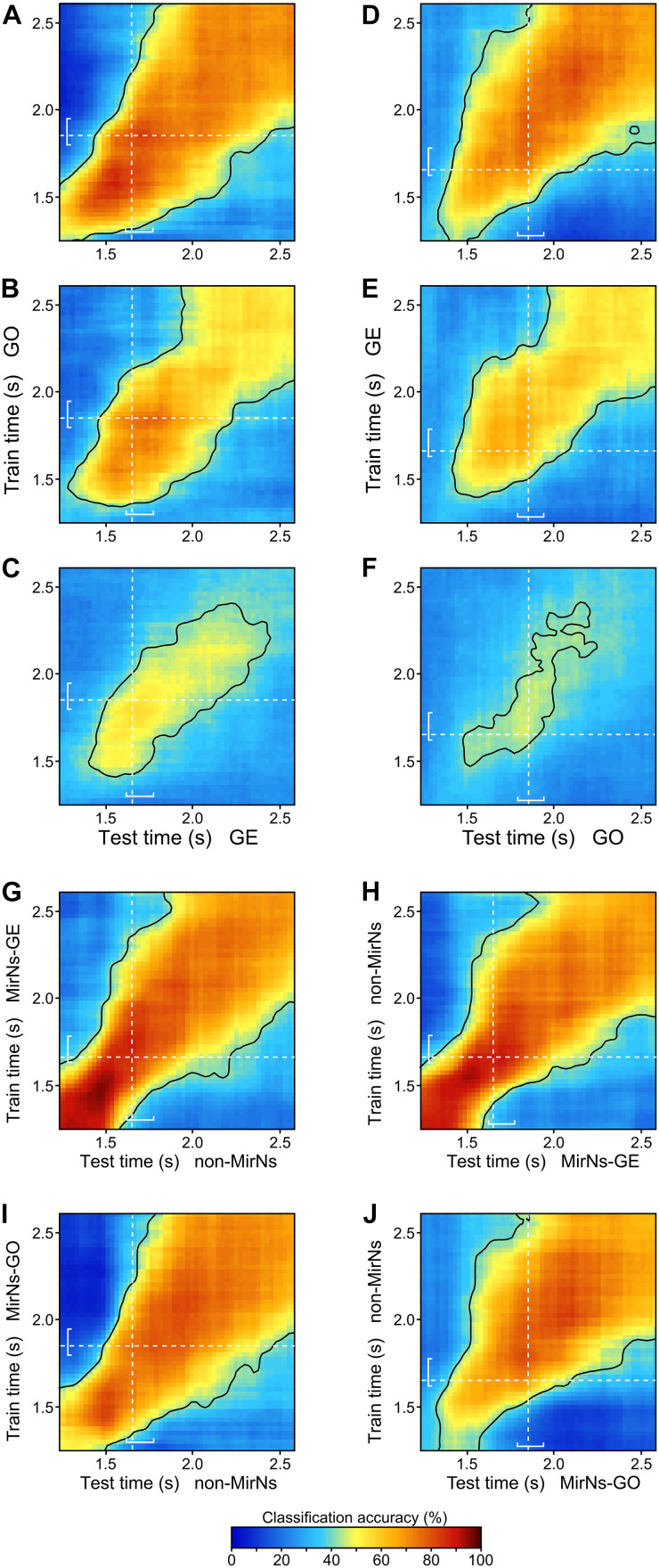
Classification in canonical space generalizes across conditions and populations. Classification was performed in the CCA-defined space (10 canonical dimensions). Cross-temporal classification of grasp configuration across (**A** to **F**) MirN execution and observation activity and (**G** to **J**) MirN and non-MirN populations. Directions: [(A) to (C)] MirNs-GO → MirNs-GE; [(D) to (F)] MirNs-GE → MirNs-GO; (G) MirNs-GE → non-MirNs; (H) non-MirNs → MirNs-GE; (I) MirNs-GO → non-MirNs; (J) non-MirNs → MirNs-GO. For MirNs, classification used all neurons [(A) and (D)], the 67 congruent neurons [(B) and (E)], or 67 randomly selected incongruent neurons [(C) and (F)]. Black contours mark significant classification (nonparametric permutation test; 500 label shuffles; one-sided *P* < 0.002). Time aligned to movement onset (1.25 s); dashed lines mark movement end.

We then evaluated cross-condition decoding within the CCA-defined space (10 canonical dimensions). In this space, cross-conditional decoding was significant during most of the movement and hold epochs in both directions (GO to GE and GE to GO) (Fig. 6, A and D). When the CCA space was constructed using incongruent neurons alone, decoding remained above chance (Fig. 6, C and F), although accuracy was lower than in the space defined by congruent neurons (Fig. 6, B and E). Together, these complementary analyses indicate that execution and observation share partially overlapping but structured population geometry.

Confusion matrices further characterized this generalization (fig. S4). In the original space, cross-conditional predictions distinguished finger prehension, whole-hand prehension, and precision grip, whereas hook grip was not reliably discriminated from the other configurations (fig. S4, A and F). Projection into the shared manifold and into the CCA-defined space reduced these misclassifications and improved differentiation among all four grasp configurations, consistent with partially overlapping but structured execution-observation geometries (fig. S4, B, C, G, and H).

Last, to examine whether MirN and non-MirN populations share aligned grasp-related dimensions, we applied CCA across neuronal populations. MirN-GE and non–MirN-GE population spaces contained aligned dimensions associated with grasp-related variables, and both populations differentiated among configurations (Fig. 4, B and E). Cross-population decoding between MirN-GE and non–MirN-GE was significant during the movement and hold periods in both directions (Fig. 6, G and H). Alignment was also observed between MirN-GO and non–MirN-GE spaces, further supporting shared grasp-related geometry across premotor populations despite differences in single-neuron response properties (Figs. 4, C and F, and 6, I and J).

### Encoding of action kinematics

We next examined whether MirN population activity contains structured information related to the kinematic organization of both observed and executed actions. Because grasping unfolds dynamically over time, grasp configuration can be characterized by the evolution of kinematic variables rather than by static end states. For both action execution and observation, we recorded the 3D Cartesian position and velocity of the index finger, thumb, and wrist, as well as grip aperture and its rate of change, for both the monkey and the human actor (fig. S5, A and B). Together, these variables provided a multidimensional description of hand transport and grip formation.

**Fig. 7.**
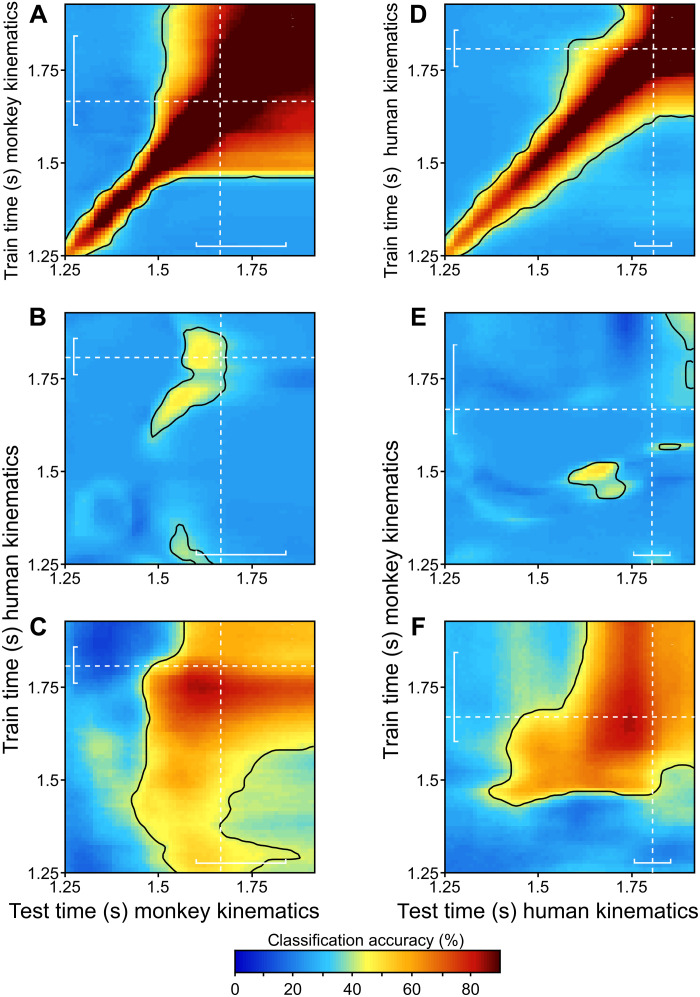
Grasp configuration classification from kinematics within and across agents. (**A** and **D**) Cross-temporal classification using monkey (A) and human (D) kinematics. (**B** and **E**) Cross-agent classification (human → monkey; monkey → human). (**C** and **F**) Cross-agent classification in CCA-defined kinematic space. Black contours indicate significant classification (nonparametric permutation test; 500 label shuffles; one-sided *P* < 0.002).

Several kinematic parameters exhibited distinct temporal profiles across grasp configurations (fig. S5C), indicating systematic differences in movement structure. Cross-temporal decoding within the kinematic space showed reliable discrimination among grasp configurations in both monkey and human datasets. Maximal discriminability occurred when training and testing times were aligned, with limited generalization during the evolving phase of movement that progressively increased toward movement completion (Fig. 7, A and D). Thus, kinematic structure itself exhibited time-dependent organization.

To quantify the relationship between neural population activity and movement kinematics, we applied multiple linear regression (MLR) analyses in both directions. Neural activity was first reduced in dimensionality using PCA, and the retained neural components were used to predict individual kinematic parameters over time. Conversely, kinematic PCs were used to predict neural activity components. All models were evaluated using cross-validation on held-out data.

**Fig. 8.**
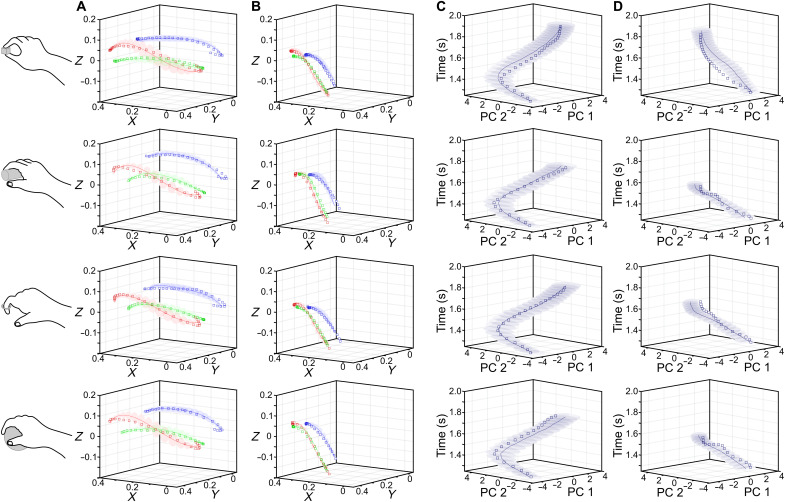
Neural activity and kinematics are mutually predictive. Regression models were fit on 100-ms binned data; trajectories are displayed at 12-ms resolution for visualization. (**A** and **B**) Model-predicted and measured kinematic trajectories of the experimenter (A) and the monkey (B). Colored markers represent predicted mean positions of index finger (red), thumb (green), and ulnar styloid (blue). Solid lines and shaded areas indicate measured mean ± SD across trials. Axes: *X* (front-back), *Y* (right-left), and *Z* (up-down). (**C** and **D**) Model-predicted and measured neural trajectories along the first two PCs during observation (C) and execution (D). Gray lines show measured mean trajectories; shaded regions indicate SD. Axes: *X* and *Y* correspond to the first and second PCs; *Z* represents time in seconds.

These analyses revealed a consistent bidirectional relationship between MirN activity and kinematic variables, reflected in high cross-validated *R*^2^ values across parameters and components (Fig. 8 and tables S2 and S3). Regression performance was higher during observation than execution, consistent with additional nonkinematic influences during movement execution.

To determine whether regression-predicted signals preserved grasp-related structure, we performed decoding analyses on predicted data. Classifiers trained on real kinematic data successfully discriminated grasp configurations from kinematics predicted from neural activity. Conversely, classifiers trained on neural data discriminated grasp configuration from neural activity predicted from kinematics (fig. S6, A, B, D, and E). Successful decoding in both directions indicates that the regression models captured structured task-related variance rather than nonspecific temporal covariation.

The neural-kinematic relationship was temporally specific. Regression models trained on progressively shorter kinematic segments showed that a 200-ms window early in the movement was sufficient to generate neural predictions that retained grasp discriminability (fig. S7), whereas temporally mismatched segments were substantially less informative. This temporal specificity reduces the likelihood that the observed relationship reflects global time-dependent structure alone.

We further examined trial-by-trial correlations between firing rates and movement duration. A large proportion of MirNs [GE: 223 of 240 (93%); GO: 189 of 240 (79%)] exhibited significant time-resolved correlations with movement duration. Population-level analyses confirmed systematic relationships between discharge and temporal aspects of movement in both execution and observation (fig. S8, A and B).

To assess whether these representational properties were specific to MirNs, we performed analogous analyses in the non-MirN population. Non-MirNs exhibited similarly strong bidirectional neural-kinematic relationships, significant classification from regression-predicted signals, and robust correlations with movement duration in most of the units [121 of 129 (94%)] (tables S2 and S3 and figs. S6, C and F, and S8C), indicating that structured kinematic information is a broader feature of premotor populations.

We next asked whether this neural-kinematic relationship resides within the shared execution-observation structure identified earlier. After restricting premotor activity to the low-dimensional shared subspace jointly defined across conditions, neural activity continued to predict kinematic variables while preserving the representational structure of grasp configurations (fig. S6, G and H). This finding indicates that kinematic information is embedded within the shared execution-observation manifold rather than confined to condition-specific dimensions.

**Fig. 9.**
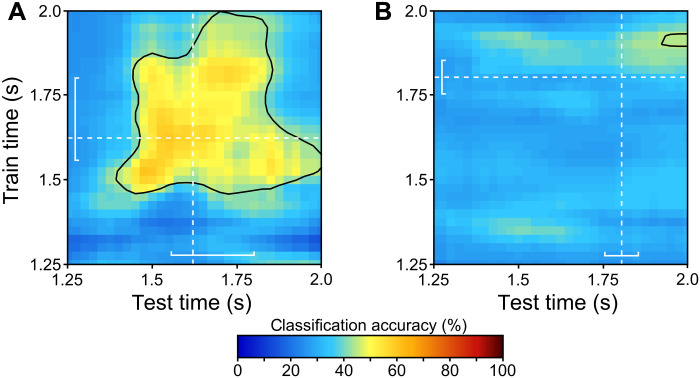
Cross-condition regression generalization. Regression coefficients derived in one condition were applied without retraining to neural data from the other condition. Classification of grasp configuration was performed by training on measured kinematic data and testing on cross-predicted kinematics (**A**) Monkey kinematics predicted from MirN execution activity using a model trained on observation activity and human kinematics. (**B**) Human kinematics predicted from MirN observation activity using a model trained on execution activity and monkey kinematics. Black contours mark significant classification (nonparametric permutation test; 500 label shuffles; one-sided *P* < 0.002).

Within this shared space, we tested cross-agent neural-kinematic generalization. A regression model trained to predict monkey kinematics from premotor activity during execution was applied, without retraining, to observation-related activity to predict human kinematics. The converse analysis was also performed. Generalization was asymmetric: Models trained on observation activity successfully predicted monkey kinematics, significantly preserving the structure of grasp configurations, whereas models trained on execution activity did not generalize to observation data (Fig. 9). This asymmetry is consistent with partially overlapping but nonidentical mappings between neural activity and kinematics across conditions.

We then examined whether kinematic representations themselves were aligned across agents. Because both monkey and human performed the same actions, their kinematic profiles were expected to exhibit systematic correspondence. Cross-agent decoding of grasp configuration from kinematic data revealed significant generalization between human and monkey movements, emerging during mid-to-late phases of movement (Fig. 7, B and E). This temporal profile paralleled the execution-observation alignment observed at the neural level. CCA applied across agents’ kinematic datasets identified aligned dimensions carrying matching grasp-related structure. Cross-agent decoding was significant during most of the movement and hold periods in both directions (Fig. 7, C and F).

Confusion matrices revealed a pattern similar to that observed for neural activity. In the original kinematic space, cross-agent predictions reliably distinguished finger prehension and precision grip, whereas hook grip and whole-hand prehension were not reliably discriminated from the other configurations (fig. S4, D and I). Projection into aligned subspaces reduced these misclassifications and improved differentiation among all four grasp configurations (fig. S4, E and J). This pattern is consistent with partial but systematic overlap between monkey and human kinematic geometries.

Together, these results indicate that premotor population activity contains structured, temporally specific information related to the evolving kinematic organization of grasping actions. This relationship generalizes across execution and observation, across neuronal populations, and across agents. While these findings do not imply a causal representation of kinematics at the single-neuron level, they are consistent with a systematic coupling between premotor population dynamics and movement structure.

Last, to assess robustness, we replicated the core analyses separately for each animal and for each premotor area (PMd and PMv). All principal findings, including grasp decoding, cross-condition generalization, subspace alignment, and kinematic prediction, were consistently observed across both monkeys and in both cortical areas (figs. S9 and S10).

## DISCUSSION

Hamilton and Grafton (*31*) proposed that actions can be analyzed at three levels: muscle, kinematic, and goal. The muscle level concerns patterns of muscular activation, the kinematic level describes movement features in space and time, and the goal level captures the intended outcome of the action. While this framework has shaped thinking about action representation, the level at which MirNs operate remains debated.

Here, we provide evidence that MirNs in the premotor cortex carry structured information related to the time-evolving kinematic structure, across both execution and observation. While end-state configurations are represented, MirN population activity also reflects the evolving kinematic structure of actions over time. This population-level organization is well suited to support flexible representation of ongoing actions relevant for motor control, action understanding, and predictive monitoring of others’ movements.

This interpretation helps reconcile the apparent opposition between low-level grip encoding and high-level goal coding. Early studies in the PMv showed that grasping neurons encode goal-directed motor acts rather than isolated limb movements, forming a “vocabulary” of motor acts (*32*). Subsequent work demonstrated selectivity for grasp categories and motor schemata in PMv (*33*, *34*) and for grip type and wrist orientation in PMd during planning and execution (*35*). Later studies emphasized goal-related encoding in PMv by manipulating action context or outcome while holding grip constant (*12*, *36*). However, when grip type is varied, grip-selective responses dominate MirN activity. Bonini *et al.* (*37*) reported grip selectivity in 70.9% of PMv neurons, whereas neurons exclusively encoding goals were rare (12.8%). Our findings are consistent with this evidence, indicating that grip and goal information often coexist and that grip-related structure is a pervasive feature of MirNs. The key question is not whether grip information is present, but how it is dynamically organized across execution and observation.

Recent work challenges a purely goal-based account of MirN function. While seminal studies by Umiltà and colleagues (*20*, *38*) shaped the goal-centered view, converging psychophysical (*39*–*41*), neurophysiological (*15*, *16*, *23*, *42*), and modeling studies (*43*) indicate that many MirN responses can be explained by shared kinematic or sensorimotor features between executed and observed actions.

Our decoding analyses reveal that grasp-related information is encoded by a dynamically reconfigured MirN ensemble rather than a fixed subset of neurons. Grasp representations emerge from transiently recruited units whose selectivity shifts across task phases. This distributed and partially redundant code supports robust, time-sensitive representation of action kinematics, enabling sensitivity to critical movement landmarks and facilitating anticipation of upcoming events. Such temporally evolving population codes reflect a broader principle of cortical computation rather than a feature unique to the motor system. Consistent with this view, time-varying population dynamics rather than sustained activity of fixed neurons underlie working memory maintenance in prefrontal cortex (*44*–*47*), highlighting the generality of dynamic coding across cognitive domains.

Regression analyses further demonstrated a strong bidirectional relationship between MirN activity and multidimensional kinematic parameters. MirN activity was systematically related to digit and wrist positions and velocities, grip aperture, and its rate of change during both execution and observation. These relationships enabled reliable decoding of grasp configuration from regression-predicted neural or kinematic data, even when models were trained on brief early movement segments. Trial-by-trial correlations with movement duration are consistent with a role for MirNs in tracking the temporal structure of action trajectories. We use the term “encoding” in an operational sense, referring to structured information captured by cross-validated models rather than implying a causal or mechanistic representation of kinematics.

The neural-kinematic relationship remained evident when premotor activity was restricted to the low-dimensional subspace shared between execution and observation, indicating that the kinematic structure is embedded within the common execution-observation geometry rather than confined to condition-specific dimensions. When cross-agent generalization was examined within this shared space, the transfer of neural-kinematic mappings proved asymmetric: Observation-derived mappings generalized to execution, whereas execution-derived mappings did not transfer to observation. This asymmetry suggests that execution-related activity incorporates additional components beyond those shared with observation, consistent with a partially overlapping representational geometry. Thus, premotor population dynamics are consistent with structured kinematic information organized within a shared but not fully symmetric execution-observation manifold.

By encoding the temporal flow of kinematics, MirNs may contribute to bridging how an action is performed with why it is performed. Early kinematic cues allow observers to infer intentions (*48*, *49*), with intention embedded in continuous movement structure (*24*, *50*). Specific kinematic features predict intention (*51*), enable action prediction without context (*52*), and dynamically influence motor excitability (*53*, *54*). Symbolic meaning and kinematics jointly shape motor responses (*55*), and informative kinematic cues can override prior expectations (*56*).

Neuroimaging, electrophysiological, and stimulation studies further support premotor sensitivity to kinematics. PMv and PMd preferentially respond to biologically plausible motion trajectories (*57*) and observed movement errors (*58*). Transcranial magnetic stimulation, magnetoencephalography, and electroencephalography studies show that motor excitability and beta-band activity track instantaneous movement parameters during observation (*59*–*62*), while point-light displays evoke robust premotor activation (*63*, *64*). Disruption of PMv function impairs grip shaping and velocity control (*65*, *66*), underscoring its contribution to kinematic processing.

Our results also clarify how execution-observation matching emerges. Although congruency was limited at the single-neuron level, population analyses revealed partially overlapping execution and observation subspaces within MirN ensembles. Neural trajectories for the two conditions evolved within shared low-dimensional structures, and cross-conditional decoding supported reliable classification of grasp configuration across conditions, particularly during mid-to-late movement phases. Alignment was not all-or-none. Cross-condition performance was strongest in subspaces enriched by neurons with a similar response structure across conditions, yet neurons classified as incongruent still contributed a weak but measurable shared structure when activity was projected into aligned components. Thus, execution-observation matching is best understood as a graded population-level phenomenon rather than a binary property of strictly congruent units.

This graded perspective helps reconcile why some studies emphasize shared coding (*14*, *29*, *67*, *68*), whereas others report condition-specific representations (*28*, *69*). Alignment does not require identical responses across neurons or complete representational symmetry; instead, it emerges dynamically from the structured geometry of population activity and coexists with condition-specific components. Such an organization naturally gives rise to asymmetric generalization while preserving shared informational content. Symmetric cross-agent generalization is not required by a partially overlapping representational geometry and would only be expected under full equivalence between execution and observation spaces. In our data, the temporal profile of shared neural coding paralleled periods of greater cross-agent kinematic alignment. Although the present study does not directly assess behavioral performance, this correspondence is broadly consistent with evidence that similarity between observed and executed movements can facilitate action understanding (*70*–*72*). Under this view, incongruency at the single-neuron level and alignment at the population level are not contradictory findings but reflect different scales of representation within the same dynamical system.

This representational congruence has implications for intracortical brain-machine interfaces (iBMIs), where observation-elicited activity could support decoder training. Observation-based calibration has been successfully used in animal models (*73*–*75*) and clinical studies (*76*–*80*) to achieve multidimensional prosthetic control. However, controlling effectors with many degrees of freedom remains challenging (*81*), and performance depends on the representational properties of recorded neuronal populations (*82*). Our findings suggest that shared, grasp-specific codes and regression-predicted kinematics provide a principled basis for improving decoder generalization and calibration efficiency.

Last, our results support a predictive role for MirNs. By encoding continuously evolving hand configurations, MirNs may contribute to detecting deviations from expected trajectories, supporting real-time motor updating and action evaluation. This view aligns with models proposing that MirNs implement forward model–like computations integrating sensory evidence with motor priors (*83*, *84*) and with evidence that MirNs encode visual feedback during both executed and observed actions (*85*).

Several limitations should be acknowledged. Recordings were confined to premotor areas in macaques, and actions were highly controlled. In addition, neurons were recorded in separate sessions rather than simultaneously, and decoding analyses relied on pseudopopulations constructed across sessions. Although this approach is widely used and enables population-level inference, it does not capture trial-by-trial correlations or noise correlations that may influence simultaneously recorded ensembles. Furthermore, kinematic measurements and neural recordings were obtained in separate sessions under matched task conditions rather than simultaneously, which precludes direct trial-by-trial alignment between neural activity and movement parameters.

The experimental design also did not dissociate action kinematics from object identity by varying grasp configuration for the same object across trials, which limits a direct test of whether premotor activity reflects kinematics independently of object-specific or goal-related factors. Although grasp configuration was not dissociated from object identity in the present design, several aspects of our findings argue against a purely object-driven account. Cross-agent generalization depended on alignment of movement kinematics across monkey and human despite differences in object appearance, and kinematic variables alone supported cross-condition and cross-agent generalization within shared population manifolds. Consistent with this interpretation, our previous work using representational similarity analysis showed that during action observation, kinematic features account for substantially more variance in MirN activity than object features (*15*). Nevertheless, future experiments explicitly dissociating object identity from grasp configuration would provide a more definitive test.

Our analyses reveal strong neural-kinematic coupling but do not establish causality, and execution-observation congruence was assessed only within the animals’ motor repertoire. Future work should extend recordings to additional cortical areas, incorporate more naturalistic behaviors, and apply causal perturbations. Translational studies should test whether these representations improve iBMI performance in closed-loop settings.

In summary, our findings support a model in which premotor population activity reflects evolving kinematic states within dynamically organized population ensembles across both execution and observation. Kinematic structure is a pervasive feature of premotor grasp-related neurons, while execution-observation alignment emerges through partially overlapping population geometries rather than through strictly congruent single neurons. Crucially, execution-observation matching is not a binary property but a graded population-level phenomenon arising from the low-dimensional organization of neural activity. Within this structured geometry, population dynamics integrate grasp configuration, movement trajectories, and cross-condition relationships over time. By implementing forward model–like computations (*86*, *87*), in a distributed and temporally evolving manner, premotor ensembles operate at the interface of motor control and action perception, linking visual input to predictive motor representations.

## MATERIALS AND METHODS

### Subjects and electrophysiological recordings

Neural recordings were obtained from two adult female rhesus macaque monkeys (*Macaca mulatta*), purpose-bred by authorized European suppliers (Deutsches Primatenzentrum, Göttingen, Germany). All procedures were conducted in a licensed facility (EL91-ΒΙΟexp-06/18-2-2014), approved by the veterinary authorities of the Region of Crete (6157/7-5-2014), and complied with European Directive 2010/63/EU (and its amendments) and Greek national legislation (Presidential Decree 56/2013) for the use of animals in research.

Under aseptic conditions and general anesthesia, a head post and a recording chamber were surgically implanted over the left hemisphere. Postoperative analgesia and antibiotics were administered, and animals were monitored daily. Extracellular recordings were performed using single tungsten microelectrodes (impedance of 0.5 to 1.5 MΩ) advanced into the cortex using a manual microdrive. Recordings targeted PMd and PMv, localized by stereotactic coordinates and anatomical landmarks (arcuate, central, and principal sulci) visible through the dura under a surgical microscope (Fig. 1).

Neural signals were amplified, bandpass-filtered (300 Hz to 5 kHz), and digitized at 25 to 30 kHz. Spike sorting was performed offline using waveform templates and PC-based clustering. Only well-isolated units with a consistent waveform throughout the recording session were included.

Spike trains of each neuron were aligned to two events: light-emitting diode (LED) illumination/object presentation (*t* = 0 s) and movement onset (*t* = 1.25 s), introducing a discontinuity at *t* = 0.8 s due to concatenation of event-aligned windows. For LED-aligned activity, spike trains were analyzed from 500 ms before to 800 ms after the event; for movement-aligned activity, analysis windows extended from 600 ms before to 1450 ms after movement onset. These two periods were concatenated to compute a discharge frequency for each trial. Discharge frequencies were calculated using 100-ms sliding windows advanced in 25-ms steps, producing 157 bins per 4-s trial.

Neuronal response timing varied across premotor cortical sectors: In some neurons, peak activity followed movement onset, whereas in others, it preceded it. To define an epoch centered on each neuron’s peak response for each condition, we applied a thresholding method. Baseline activity was first estimated from the 500-ms preceding trial onset. A movement modulation epoch was then identified, consisting of at least seven consecutive firing rate samples (200 ms), beginning no earlier than 450 ms before movement onset and ending no later than 600 ms after the end of the holding period, with activity exceeding the baseline average plus one SD. Neuronal modulation was assessed using a two-way analysis of variance (ANOVA) followed by Bonferroni post hoc tests, with factors epoch (baseline versus movement modulation epoch) and grasp configuration (four levels). Neurons were considered significantly modulated if activity during the movement modulation epoch differed from baseline (*P* < 0.05), provided that the mean firing rate exceeded 10 spikes/s during the movement high-activity epoch for at least one grasp configuration. These analyses were performed separately for execution and observation conditions. Neurons showing task-related modulation during both execution and observation were classified as MirNs, whereas neurons modulated only during execution were classified as non-MirNs.

### Behavioral task

Monkeys were trained to sit in a primate chair with their head fixed. A rotating, uniform hexagonal prism was positioned in front of the monkey. Each of its six perpendicular rectangular faces accommodated a 3D object mounted on a metal rod passing through the face center, allowing slight horizontal displacement (<0.3 cm) upon grasping. A microswitch on each rod signaled object holding. The apparatus was placed 25 cm from the monkey during execution trials (intrapersonal space) and 50 cm during observation trials (extrapersonal space). One object was presented per trial in a fixed central position (fig. S1).

Four objects were used, each associated with a specific, explicitly trained grasp configuration: a large sphere (40-mm diameter), grasped with whole-hand prehension involving palm contact; a cylinder (40-mm length, 20-mm base diameter), grasped with finger prehension excluding the thumb; a ring (15-mm diameter), grasped with a hook grip involving index finger insertion; and a cube positioned in a vertical groove (10-mm side), grasped with an advanced precision grip using opposed pulpar surfaces of the distal phalanges of the index finger and thumb. Following an extensive training period (6 to 8 months), the animals’ grasping behavior reached a stable plateau characterized by highly stereotypical and reproducible grip patterns. For each object, a single grasp configuration archetype was reinforced throughout the experiment, and both monkeys adopted comparable grasp configurations. Grasp performance was continuously video-monitored to ensure adherence to the instructed grasp configuration. During the recording phase, execution remained uniform across sessions, with no trials requiring exclusion due to grasp errors. Motion capture recordings confirmed that each object was associated with a statistically distinct and reproducible kinematic pattern with minimal trial-to-trial variance (fig. S5). Eye movements were recorded with the scleral search coil technique at 500 Hz.

GE trials began with illumination of an LED above the object. The monkey fixated the LED and pressed a lap-level button for 800 to 1200 ms. A brief LED flash cued button release and initiation of the reach-to-grasp movement while maintaining fixation. The monkey grasped and held the object with the right hand for 600 to 900 ms; fixation was enforced within a 10°-diameter window centered on the object. LED offset signaled object release. Correct trials were rewarded with water.

During GO trials, the monkey remained immobile and observed the experimenter performing the execution task. Hand immobility was verified by continuous video monitoring; trials with forelimb movements were discarded. The experimenter stood to the right of the animal and grasped the same objects with the right hand. The LED remained off, and the experimenter received instructions from a screen not visible to the monkey. During the observation task, oculomotor behavior was not explicitly constrained, consistent with most electrophysiological studies of MirNs, to preserve natural viewing behavior. Nevertheless, eye position was continuously recorded to verify that the animals attended to the presented action. To quantify visual engagement, we measured the proportion of observation trials in which gaze fell within a 10°-diameter window centered on the object before hand-object contact. This criterion was met in more than 95% of trials, consistent with our previous findings (*16*), indicating that the animals reliably fixated the relevant region during the critical phase of the observed movement. During the observation task, animals received a reward at the end of each trial identical in amount to that delivered during execution trials, to eliminate potential confounds related to reward expectancy or delivery.

Tasks were performed under ambient lighting of normal intensity in blocks of up to 10 trials for each of the four reach-to-grasp actions, with observation blocks typically preceding execution blocks. In both tasks, movement onset and offset (monkey during execution, experimenter during observation) were marked by button release and activation of the object displacement switch, respectively.

### Recording of kinematics

Reaching-to-grasp kinematics were recorded using a V120:Trio OptiTrack motion capture system (NaturalPoint Inc., USA) controlled by Motive software, sampling at 120 Hz. These recordings were obtained in sessions separate from the neuronal recordings under identical task conditions. The camera bar was positioned to fully cover the 3D workspace during both GE and GO. Precalibrated sensors and a fixed three-axis coordinate frame eliminated the need for user calibration.

Three lightweight reflective markers were attached to the dorsal surfaces of the nails (distal interphalangeal joints) of the index finger and thumb and to the ulnar styloid of the wrist. Marker positions were tracked in 3D, allowing reconstruction of movement trajectories.

Kinematic data were recorded across three sessions (two monkeys and one human), each with approximately 55 repetitions per action (total *n* = 170 per action). Behavioral events (object presentation, go cue, grasp onset, and release) were logged and used for alignment.

### Population activity processing

For each neuron, trial-averaged firing rates were baseline-corrected by subtracting spontaneous activity (mean firing rate during the 600-ms pre-presentation period) and normalized to the maximum firing rate across conditions (baseline ≈ 0; peak ≈ 1). Population activity was then computed by averaging across neurons for each condition.

PCA was used to visualize and compare population dynamics. For each neuron and condition, trial-averaged grasp-specific firing rates were concatenated, producing two matrices (GE and GO) of size (neurons) × (4 grasps × time bins). PCA was performed separately for GE and GO. Proportion of explained variance for each component was computed for both within and across condition projections. An alignment index was estimated for across condition projections and compared to a randomized subspace according to the method described by Elsayed *et al.* (*30*). This procedure was applied in a cross-validated way. Trials per each grip and condition were split in two halves, and one of them was used for calculating the PCA space while the other was used for projecting and calculating explained variances and the alignment index. Cross-projections were also done using size matched subsets of trials from the two conditions.

### Decoding analyses

To assess grasp information in neural populations, we used a Poisson naïve Bayes classifier [Neural Decoding Toolbox; (*88*)]. A Poisson naïve Bayes classifier is a specialized version of naïve Βayes classifier, in which class conditional probabilities are modeled with the Poisson distribution. Because neurons were recorded in separate sessions, pseudopopulations were constructed by concatenating single-trial data across neurons (*45*).

For each neuron (MirNs: *n* = 240, non-MirNs: *n* = 129), nine trials per grasp configuration and condition were randomly selected, yielding 36 samples per time bin per condition. Leave-one-out cross-validation was performed nine times (training: 32 trials, 8 per grip; testing: 4 trials, 1 per grasp configuration), and this process was resampled 100 times to smooth results.

Time-resolved decoding trained and tested the classifier on the same bin. Cross-temporal decoding trained on one time bin and tested across all bins, producing 2D accuracy maps.

Cross-conditional decoding trained on one condition (GE or GO) and tested on the other, using all neurons (*n* = 240), only congruent neurons (*n* = 67), or a size matched set of incongruent neurons (*n* = 67) (see the “Congruence index” section).

Classification accuracy was defined as the percentage of correct predictions out of the total number of predictions. Significance was determined with a permutation test by shuffling trial labels and repeating the decoding process 500 times to generate null distributions; decoding exceeding all shuffled runs was considered significant (*P* < 0.002, one-sided).

### Decoding with most selective neuron subsets

To assess the patterns of neuron recruitment across different time points, we performed an analysis excluding the 120 most selective neurons (precomputed *P* values; see the “Single-unit selectivity” section) at a chosen time bin and evaluated how their removal affected decoding across other time bins. We repeated this analysis for all bins. To determine significance, we ran the same decoding analysis 500 times, excluding 120 randomly selected neurons, creating a distribution of decoding accuracies for each time bin. We *z*-scored decoding performance relative to the random removal distribution and calculated one-sided *P* values to test whether the removal of the most selective neurons significantly reduced decoding performance compared to the removal of random neurons. The significance level was set at *P* = 0.01. For non-MirNs, the same analysis was performed subtracting 65 neurons, which corresponded to half of the population.

To evaluate the role of the most selective neurons, we ranked units by grasp selectivity (one-way ANOVA on training data; factor: four grasp configurations; *n* = 32; eight trials per grasp configuration; units ranked by *P* values) at each time bin, following the Neural Decoding Toolbox’s pipeline (*88*). Decoding was performed using the following: (i) only the 80 most selective neurons, (ii) neurons ranked 81 to 160, or (iii) excluding the top 160 neurons at each time bin. Only diagonal (train = test) decoding was performed. For non-MirNs, we used three groups of 43 neurons to perform the same analysis.

### Single-unit selectivity

For each neuron and time bin, grasp selectivity was tested using a one-way ANOVA (factor: four grasp configurations; *n* ≥ 36; ≥9 trials per grasp configuration; *P* < 0.05 for ≥7 consecutive bins), producing a (neurons × time) matrix of *P* values. Neurons were sorted according to the time of their first selective bin, excluding selectivity periods that ended more than 250 ms before movement onset.

### Selectivity stability index

We computed a selectivity stability index (SI) to quantify how consistently neurons maintained their grasp configuration preference across time. For each neuron, grasps were ranked by firing rate at each selective bin, and SI was calculated for each time pair

$$\text{SI}\left(t_{1},t_{2}\right)=\frac{\sum_{i=1}^{4}w_{i,t_{1}}\left(1-|R\left(i,t_{1}\right)-R(i,t_{2})|\right)}{\sum_{i=1}^{4}w_{i,t_{1}}}$$

where $w_{i,t}=1-\frac{R\left(i,t\right)-1}{4}$, i∈[1,4], $R_{i,t}$ is the rank of grasp *i* at time *t*, and $w_{i,t}$ is a weight that prioritizes the stability of higher-ranked grasps. Thus, this index captures changes in all grasp ranks, with an emphasis on preferred grasps. SI values range from −1 (unstable/reversed order) to 1 (stable/identical order). Positive SI values indicate stability, occurring when a neuron’s top-ranked grasp remains unchanged or when the only change is a swap between the first- and second-ranked grasps. To capture population-level stability, we summed the positive SI values across neurons and applied a soft normalization by the number of selective units at the reference time, using a constant of 12 (5% of the population) to ensure that times with very few selective neurons do not appear as highly stable.

### Congruence index

To assess matching between execution and observation, we created a selectivity congruence index (CI), computed in the same way as SI but comparing grasp rankings across conditions

$$\text{CI}\left(t_{\text{GO}},t_{\text{GE}}\right)=\frac{\sum_{i=1}^{4}w_{i,t_{\text{GO}}}\left(1-|R_{\text{GO}}\left(i,t_{\text{GO}}\right)-R_{\text{GE}}(i,t_{\text{GE}})|\right)}{\sum_{i=1}^{4}w_{i,t_{\text{GO}}}}$$

where $w_{i,t}=1-\frac{R_{\text{GO}}\left(i,t\right)-1}{4}$, i∈[1,4].

As before, to capture population-level congruence, we summed the positive CI values across neurons. We classified neurons that exhibited a high total positive CI between 100 ms before movement onset and the hold phase, across all time pairs, as congruent. This threshold corresponded to more than 150 ms × 150 ms of perfect congruence. Sixty-seven neurons (28% of the MirN population) were classified as congruent.

### Canonical correlation analysis

To test whether MirNs share the same representational code during observation and execution, we performed CCA. First, we calculated the grasp-specific trial-averaged neural trajectories separately for observation and execution. We then subtracted the corresponding condition-specific trial-averaged trajectories [subtracted average GO (GE) trajectory from GO (GE) grasp configuration–specific trajectories]. In that way, we excluded condition-specific information from our trajectories and kept only grasp configuration–specific information. We then concatenated all grasp configurations along the time dimension for each condition and created two matrices with dimensions (neurons) × (four grasp configurations × time bins). We performed PCA on each of these matrices to reduce the dimensionality and kept the 10 first dimensions. Then, we applied CCA on the resulting pair of matrices. This analysis was run in a cross-validated way by splitting trials into two halves, using one half of the trials to create the canonical space and projecting the other half of the trials on the canonical space to compute correlations between the two conditions. This was repeated 20 times. Thus, for each canonical dimension, we created a distribution of canonical correlations. To test their significance, we repeated the same procedure after shuffling the grasp configuration labels 500 times and created a null distribution. *P* values were calculated as the proportion of null correlations that were greater than the mean of the canonical correlations for each dimension (significance level *P* = 0.01). We also repeated the same analysis with data from within each condition to create upper limits for the correlations.

To perform decoding analyses in the CCA space, we used four trials per grasp configuration to compute the canonical dimensions and then projected pseudopopulation trials excluding those four trials on the found dimensions. This process was repeated 500 times. We applied cross-conditional decoding analyses as described previously using these data.

### Kinematic decoding

We trained a support vector machine (SVM) with a linear kernel, to decode grasp configuration from 20 kinematic features (wrist, thumb, and index positions and velocities in *X*, *Y*, *Z*, plus aperture and its rate of change). Ten trials per grasp configuration and per agent were sampled, yielding 40 samples per agent. A leave-one-out cross-validation (training: 36 trials, 9 per grasp; testing: 4 trials, 1 per grasp) was performed to train and test the decoder. This procedure was repeated 100 times. Decoding performance and significance were assessed in the same way as for the neural population decoding (see the “Decoding analyses” section). Decoding was performed within-agent (monkey or human) and across-agent (cross-conditional: human → monkey and monkey → human).

Across-agent decoding was also performed after applying CCA to the kinematic parameters. As described above for neuronal data, CCA was applied on two matrices that contained the five first PCs of the human and monkey kinematics respectively, with the four grasp configurations concatenated.

### Multiple linear regression

To quantify the relationship between neural activity and action kinematics, we used MLR. Kinematic data were formatted, analogously to neural data, into consecutive 100-ms bins with 25-ms sliding steps, yielding 36 time bins. The first bin started 100 ms before movement onset, and the total duration spanned 975 ms, ending ~200 ms (GO) or 600 ms (GE) after movement offset.

Kinematic data were first trial averaged separately for each grasp configuration and then concatenated across grasp configurations, resulting, for each kinematic parameter, in a time series of 36 × 4 data points per condition. Neural data were processed in the same manner, retaining only the time bins corresponding to the kinematic windows (36 bins starting 100 ms before movement onset).

Because of the high dimensionality of the neural dataset (~120 neurons), dimensionality was reduced using PCA. In some of the analyses instead of PCA, we reduced the dimensionality by projecting the neural activity on the shared subspace (see the “Shared subspace decoding” section). The first seven PCs, accounting for ~80% of the variance during this period, were retained. Neural data were thus arranged in a 7 × (36 × 4) matrix.

The model predicting kinematics from neural PCs was

$$Y_{i}\left(t\right)=\beta_{i,0}+\sum_{j=1}^{7}\beta_{i,j}X_{j}\left(t\right)+\varepsilon_{i}\left(t\right)$$

i∈[1,20], where $Y_{i}\left(t\right)$ are kinematic parameters, $X_{j}\left(t\right)$ are the neural PCs, and $\varepsilon_{i}\left(t\right)$ are the model’s residuals. Coefficients $\beta_{i,j}$ were estimated using ordinary least squares independently for each kinematic parameter. This procedure yielded predictions of each kinematic parameter from neural activity.

In addition, we used MLR to predict neural activity from kinematic parameters. Data were processed as described above, with an additional PCA applied to the kinematic data to reduce regressor dimensionality. The first five kinematic PCs were retained, yielding a 5 × (36 × 4) matrix. Neural data were represented as seven separate time series, each corresponding to one neural PC.

MLR was applied according to

$$y_{i}\left(t\right)=b_{i,0}+\sum_{j=1}^{5}b_{i,j}x_{j}\left(t\right)+\varepsilon_{i}\left(t\right)$$

i∈[1,7], where $y_{i}\left(t\right)$ are neural PCs, $x_{j}\left(t\right)$ are the kinematic PCs, and $\varepsilon_{i}\left(t\right)$ are the model’s residuals. Coefficients $b_{i,j}$ were estimated using ordinary least squares independently for each neural component. This analysis yielded predictions of each neural PC from kinematic data.

To validate the model with decoding, we only used four randomly selected trials per grasp configuration to create the kinematic and neural trial averages. This was done to avoid using the same trials for the regression model and the decoding. The *R*^2^ value for each model was calculated from held-out trials.

### Decoding with predicted data

To validate the regression models, SVM classifiers were trained on actual data (neural or kinematic) and tested on regression-predicted data. Five trials per grasp configuration, per agent, and per data type (kinematic or neural) were sampled yielding 20 samples per agent and per data type. Trials used in the regression were excluded from this sampling. A fivefold leave-one-out cross-validation (training: 16 trials, 4 per grasp; testing: 4 trials, 1 per grasp configuration) was performed to train and test the decoder. This procedure (including the regression with four trials per grasp configuration) was repeated 500 times. Decoding performance and significance were assessed in the same way as for the neural population decoding (see the “Decoding analyses” section).

Moreover, we applied cross prediction decoding analyses to test whether the neural-kinematic relationship generalized across conditions. In this case, we used nine trials per grasp configuration from GO (GE) neural and kinematic data to create the regression model. Then, we used nine trials per grasp configuration from GE (GO) neural activity and projected them using the previously calculated regression coefficients. In that way, we applied a model created from one condition to predict kinematic parameters of the other condition. To validate this generalization, we trained SVM classifiers to differentiate grasp configurations on the recorded kinematic data and tested them on the cross predicted kinematic data.

### Reduced time regression

We extended the MLR analysis for predicting neural activity by progressively reducing the time window used for regression. This approach allowed us to assess how much kinematic information is required to accurately predict neural activity up to the end of the movement. We began by removing the final 100 ms of the data and applied the MLR procedure to the remaining time window. The resulting regression coefficients were then used to project the full dataset, yielding predicted neural activity. Decoding analyses were subsequently performed on the predicted data to evaluate prediction accuracy over time. This procedure was repeated by iteratively shortening the regression window in 100-ms steps until prediction accuracy deteriorated.

### Explained variance in kinematics

We used a one-way ANOVA (factor: four grasp configurations; *n* = 477; ≥108 per grasp configuration for monkey; *n* = 201; ≥50 per grasp configuration for human) to calculate the percentage of variance explained by grasp configuration for each kinematic parameter, using ω^2^ as the effect size (*89*).

### Shared subspace decoding

Shared and exclusive subspaces for GE and GO were computed using the Manopt toolbox (*90*), following (*29*). A 12D shared subspace was identified by maximizing joint variance orthogonal to condition-exclusive subspaces. Pseudopopulation trials were projected into this subspace, and cross-conditional decoding was repeated using all neurons, congruent neurons only, or incongruent neurons only, as described in the “Decoding analyses” section.

### Correlations with movement duration

We computed time-resolved correlations between neuronal firing rates and movement duration across all recorded neurons. For each neuron, we constructed a firing rate matrix comprising all trials (number of trials × time bins) and a corresponding vector containing the movement duration for each trial (number of trials). Pearson’s correlation coefficients were then calculated between movement duration and firing rate at each time bin by correlating the movement duration vector with each column of the firing rate matrix. This procedure yielded a 2D matrix (number of neurons × time bins) containing correlation coefficients for all neurons and time bins, along with a corresponding matrix of *P* values.

To assess the significance of these correlations at the population level, correlation coefficients were transformed using Fisher’s *z*-transformation according to the equation

$$z=\frac{1}{2}\text{ln}\left(\frac{1+r}{1-r}\right)$$

where 𝑟 denotes the Pearson correlation coefficient. This transformation normalizes the skewed distribution of correlation values and enables population-level statistical inference. For each time bin, a weighted mean *z*-value was computed as

$$\bar{z}=\frac{\sum_{i}z_{i}\left(n_{i}-3\right)}{\sum_{i}\left(n_{i}-3\right)}$$

where 𝑛 is the number of trials used to compute the correlation for neuron 𝑖. Population-level significance was assessed by testing whether the mean *z* differed from zero using a two-tailed criterion (|*z*| > 1.96; *P* < 0.05) (*91*).
