# Supplementary material for: Dynamic population coding of kinematic structure across executed and observed actions in primate premotor cortex
Source: Sci Adv. 2026 Jun 19;12(25):eaed9309. doi: 10.1126/sciadv.aed9309 (PMC13281793; doi:10.1126/sciadv.aed9309)
Supplement: Supplementary file 1 — Figs. S1 to S10 Tables S1 to S3 [file sciadv.aed9309_sm.pdf]

Science Advances
AAAS

Supplementary Materials for

Dynamic population coding of kinematic structure across executed and observed actions in primate premotor cortex

Konstantinos Chatzimichail *et al.*

Corresponding author: Vassilis Raos, vasraos@gmail.com

Sci. Adv. **12**, eaed9309 (2026)
DOI: 10.1126/sciadv.aed9309

This PDF file includes:

Figs. S1 to S10
Tables S1 to S3

Fig. S1.

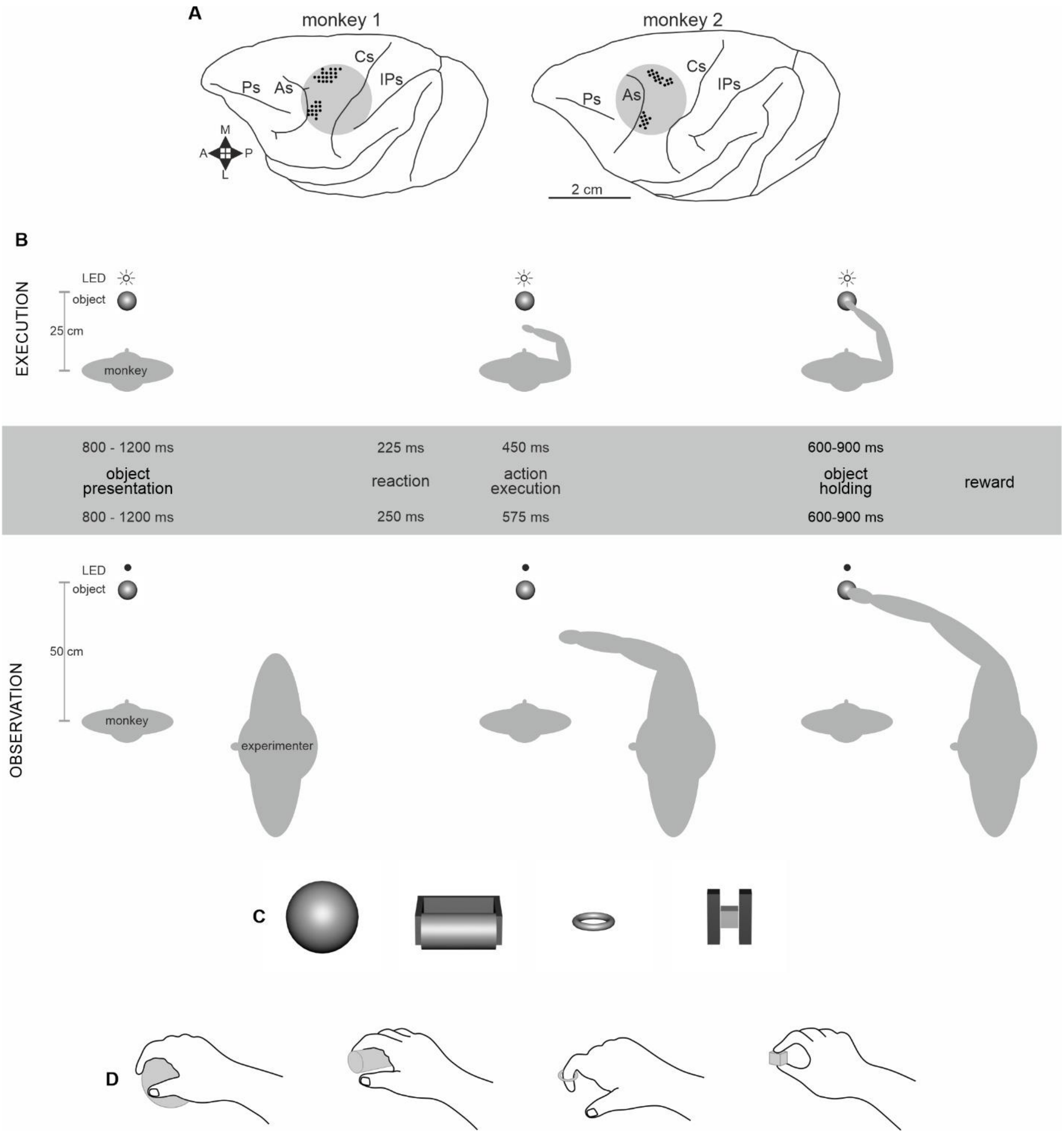

Fig. S1. Recording sites, behavioral tasks, objects, and grasp configurations. A: Schematic of the left hemispheres of the two monkeys showing recording sites in PMd and PMv. Dots indicate electrode entry points; shaded regions indicate the recording chamber locations. As, arcuate sulcus; Cs, central sulcus; IPs, intraparietal sulcus; Ps, principal sulcus; A, anterior; L, lateral; M, medial; P, posterior. **B**: Spatial arrangement of the monkey, experimenter, and objects during grasp execution (GE;

upper row) and grasp observation (GO; lower row), shown from above at object presentation (left), movement (middle), and hold (right). In GE, the object was placed in the monkey's peripersonal space (25 cm), and the monkey reached with its right hand following LED cues. In GO, the object was placed in extrapersonal space (50 cm), and the experimenter performed the reach-to-grasp action while the monkey observed without moving. Mean reaction times, movement durations, and delay and hold intervals (uniformly distributed) are indicated. **C**: Three-dimensional models of the four grasped objects. **D**: Drawings of the grasp configurations associated with each object.

Fig. S2.

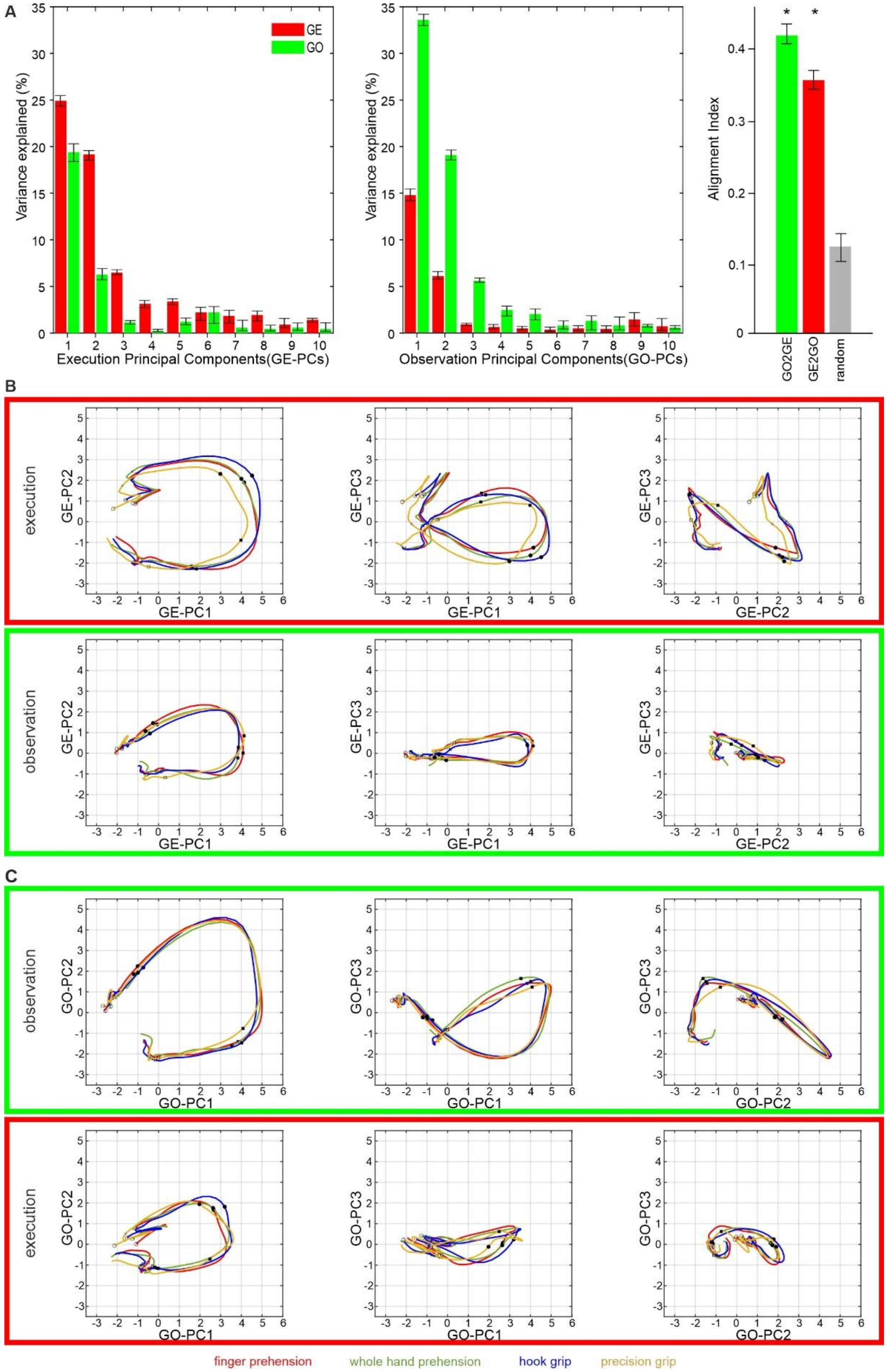
A
GE
GO
Variance explained (%)
Execution Principal Components(GE-PCs)
Observation Principal Components(GO-PCs)
Alignment Index
GO2GE
GE2GO
random
B
execution
observation
GE-PC1
GE-PC2
GE-PC3
C
GO-PC1
GO-PC2
GO-PC3
finger prehension
whole hand prehension
hook grip
precision grip

Fig. S2. Execution and observation share partially overlapping neural dimensions. Principal component analysis (PCA) was performed separately for GE and GO in a cross-validated manner. **A**: Percentage of variance explained in observation (green) and execution (red) by the top 10 principal components derived from GE (left) and GO (middle). Right panel: normalized cross-projected variance (alignment index) for GO variance in GE-PCs (green) and GE variance in GO-PCs (red), compared to randomized subspaces (gray; 95% confidence interval). Statistical significance was assessed using non-parametric permutation testing (1000 label shuffles; one-sided $p < 0.01$). **B**: Population trajectories during GE (red) and GO (green), averaged across trials of each grasp configuration, projected onto the first three principal components of GE activity. **C**: Same as in B, projected onto the first three principal components of GO activity. Symbols indicate task events (open circle: trial onset; cross: go cue; filled circle: movement onset; filled square: movement end; open square: hold end).

Fig. S3.

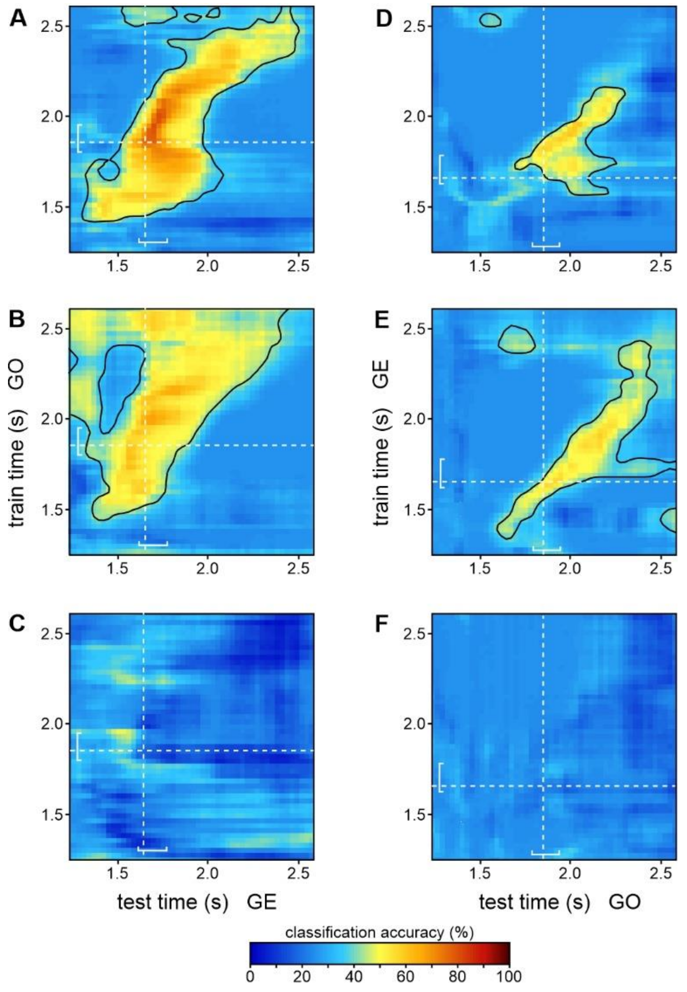

Fig. S3. Classification of grasp configuration in the shared subspace. Cross-temporal classification was performed in the shared execution-observation subspace (12 dimensions). **A-C**: GO → GE; **D-F**: GE → GO. Classification was based on all neurons (**A**, **D**), the 67 congruent neurons (**B**, **E**), or 67 randomly selected incongruent neurons (**C**, **F**). Color scale indicates classification accuracy. Black contours mark significant classification (non-parametric permutation test; 500 label shuffles; one-sided $p < 0.002$). Time aligned to movement onset (1.25 s); dashed lines indicate movement end.

Fig. S4.

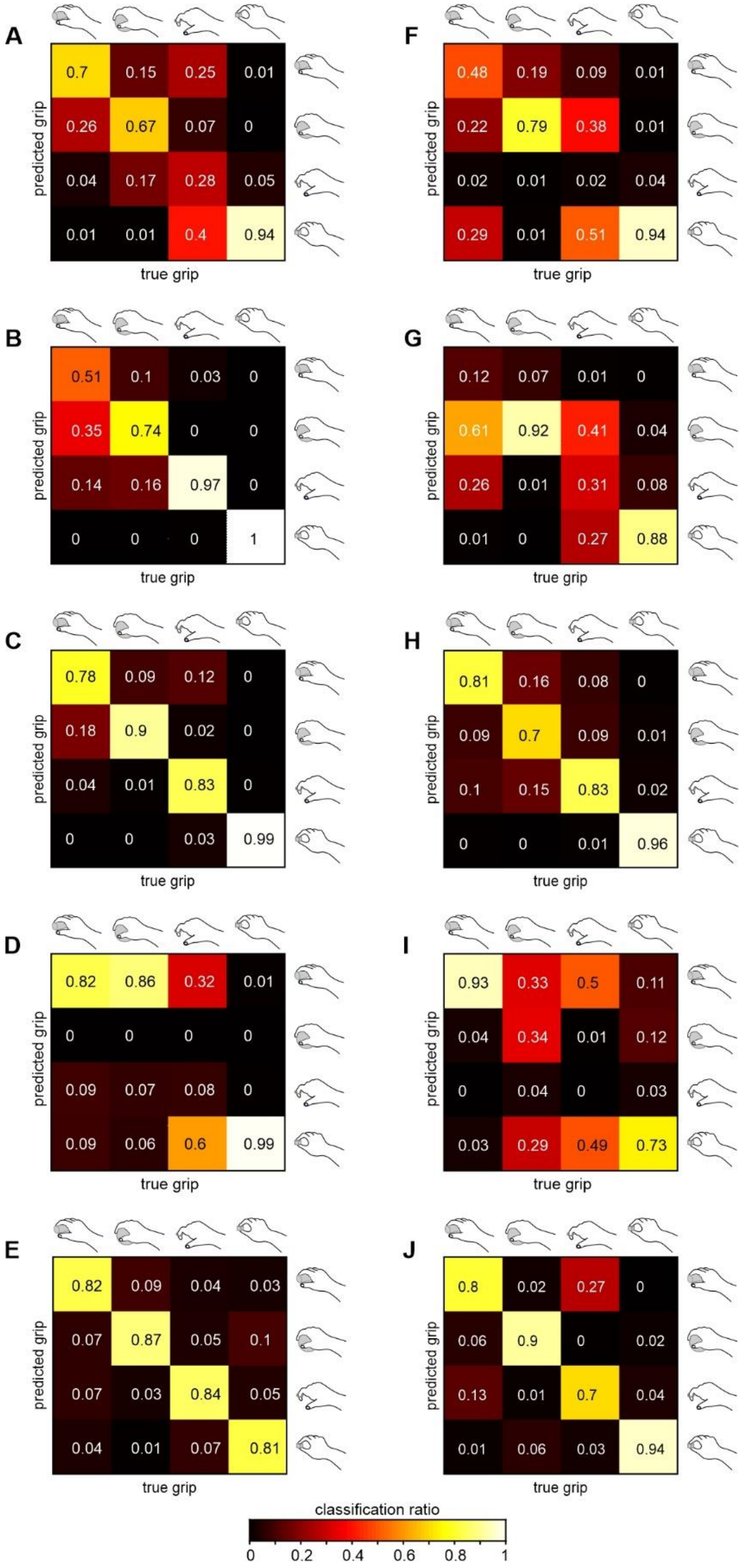

A
0.7 0.15 0.25 0.01
0.26 0.67 0.07 0
0.04 0.17 0.28 0.05
0.01 0.01 0.4 0.94
predicted grip
true grip
B
0.51 0.1 0.03 0
0.35 0.74 0 0
0.14 0.16 0.97 0
0 0 0 1
C
0.78 0.09 0.12 0
0.18 0.9 0.02 0
0.04 0.01 0.83 0
0 0 0.03 0.99
D
0.82 0.86 0.32 0.01
0 0 0 0
0.09 0.07 0.08 0
0.09 0.06 0.6 0.99
E
0.82 0.09 0.04 0.03
0.07 0.87 0.05 0.1
0.07 0.03 0.84 0.05
0.04 0.01 0.07 0.81
F
0.48 0.19 0.09 0.01
0.22 0.79 0.38 0.01
0.02 0.01 0.02 0.04
0.29 0.01 0.51 0.94
G
0.12 0.07 0.01 0
0.61 0.92 0.41 0.04
0.26 0.01 0.31 0.08
0.01 0 0.27 0.88
H
0.81 0.16 0.08 0
0.09 0.7 0.09 0.01
0.1 0.15 0.83 0.02
0 0 0.01 0.96
I
0.93 0.33 0.5 0.11
0.04 0.34 0.01 0.12
0 0.04 0 0.03
0.03 0.29 0.49 0.73
J
0.8 0.02 0.27 0
0.06 0.9 0 0.02
0.13 0.01 0.7 0.04
0.01 0.06 0.03 0.94
classification ratio
0 0.2 0.4 0.6 0.8 1

Fig. S4. Confusion matrices for cross-condition and cross-agent classification. Confusion matrices show classification ratios for grasp configuration classification. Numerical values are reported in each cell. Values correspond to the time window exhibiting peak significant classification. **A**-**C**: GO → GE in the original neural space (**A**), shared neural subspace (**B**), and CCA-defined neural space (**C**). **F**-**H**: GE → GO in the original neural space (**F**), shared neural subspace (**G**), and CCA-defined neural space (**H**). **D**-**E**: Human → monkey kinematic space in original kinematic space (**D**) and CCA-defined kinematic space (**E**). **I**-**J**: Monkey → human in original kinematic space (**I**) and CCA-defined kinematic space (**J**). Color scale indicates proportion of classifications assigned to each grasp configuration.

Fig. S5.

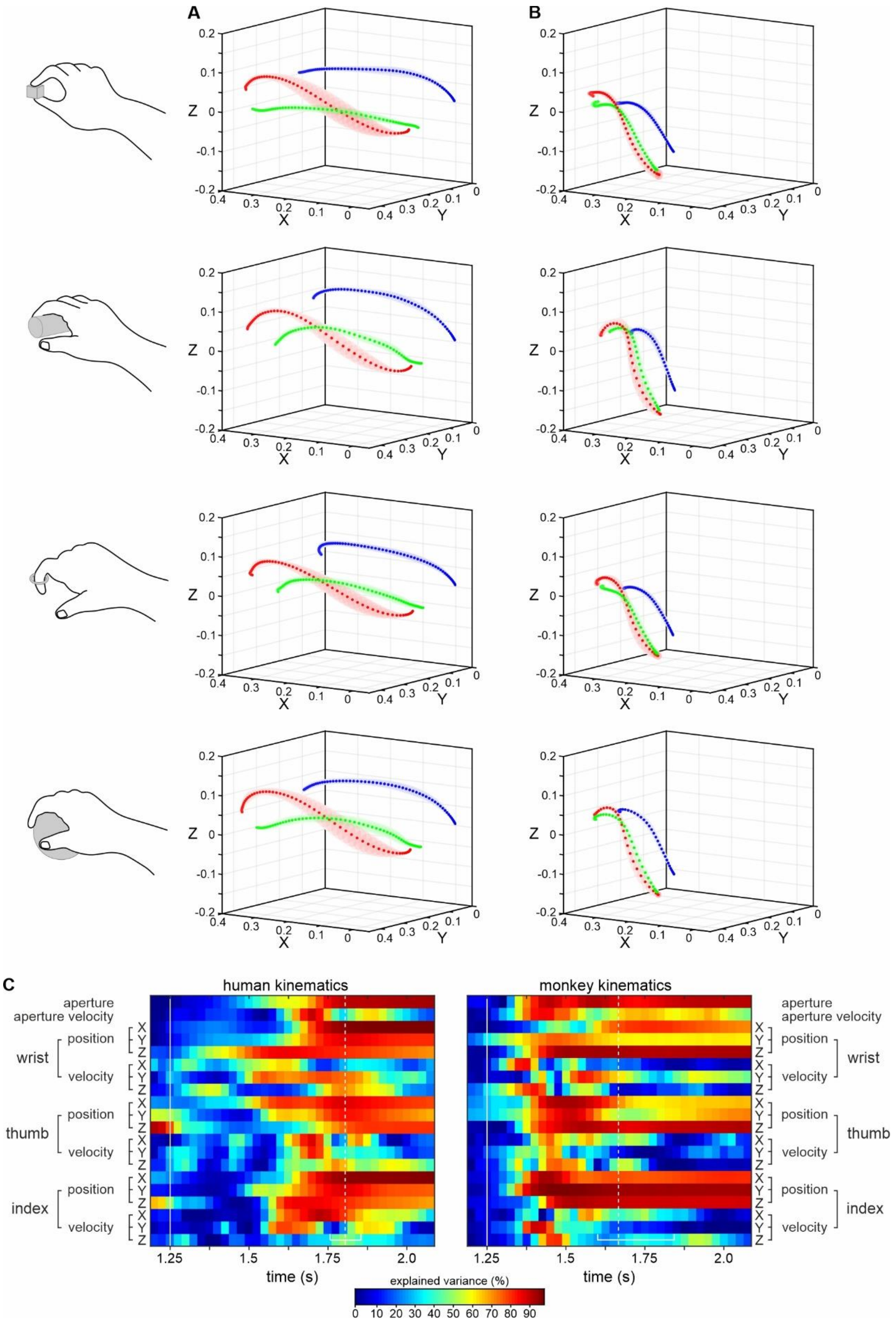
A
B
C
human kinematics
monkey kinematics
aperture
aperture velocity
wrist
thumb
index
position
velocity
X
Y
Z
time (s)
explained variance (%)
0 10 20 30 40 50 60 70 80 90
1.25
1.5
1.75
2.0

Fig. S5. Human and monkey kinematics differ systematically across grasp configurations. **A**, **B**: Reaching-to-grasp kinematics of the experimenter (**A**) and monkey (**B**) shown in Cartesian space. Red, green, and blue traces represent mean positions of the index finger, thumb, and ulnar styloid (wrist), respectively, sampled at 12 ms resolution. Shaded regions indicate standard deviation across trials. Axes correspond to X (front–back), Y (right–left), and Z (up–down). **C**: Percentage of variance explained (ANOVA ω^2) by grasp configuration for kinematic parameters, including positions and velocities of the wrist, thumb, and index finger (X, Y, Z), as well as grip aperture and its rate of change. Color bar indicates percentage of explained variance.

Fig. S6.

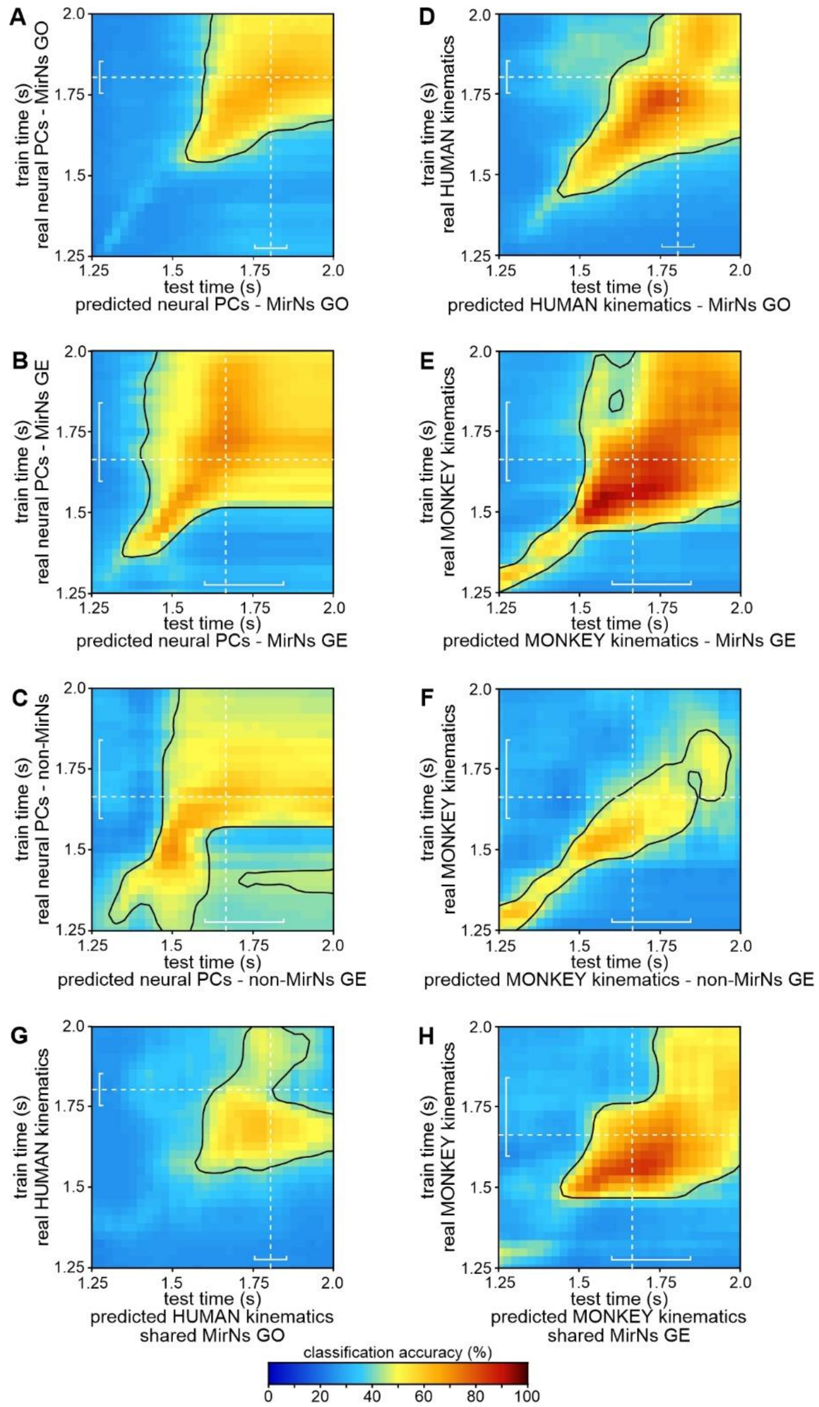

Fig. S6. Regression-predicted neural and kinematic data preserve grasp configuration information. Cross-temporal classification was performed using data generated by regression models. Classifiers were trained on measured data and tested on regression-predicted data. **A**-**C**: Neural activity predicted from action kinematics. **D**-

F: Kinematics predicted from neural activity. **A**, **D**: MirNs during GO / human kinematics. **B**, **E**: MirNs during GE / monkey kinematics. **C**, **F**: Non-MirNs / monkey kinematics. **G**, **H**: Human and monkey kinematics predicted from MirNs-GO and MirNs-GE neural activity projected onto the shared subspace, respectively. Black contours indicate significant classification (non-parametric permutation test; 500 label shuffles; one-sided $p < 0.002$). Time aligned to movement onset (1.25 s); dashed lines indicate movement end.

Fig. S7.

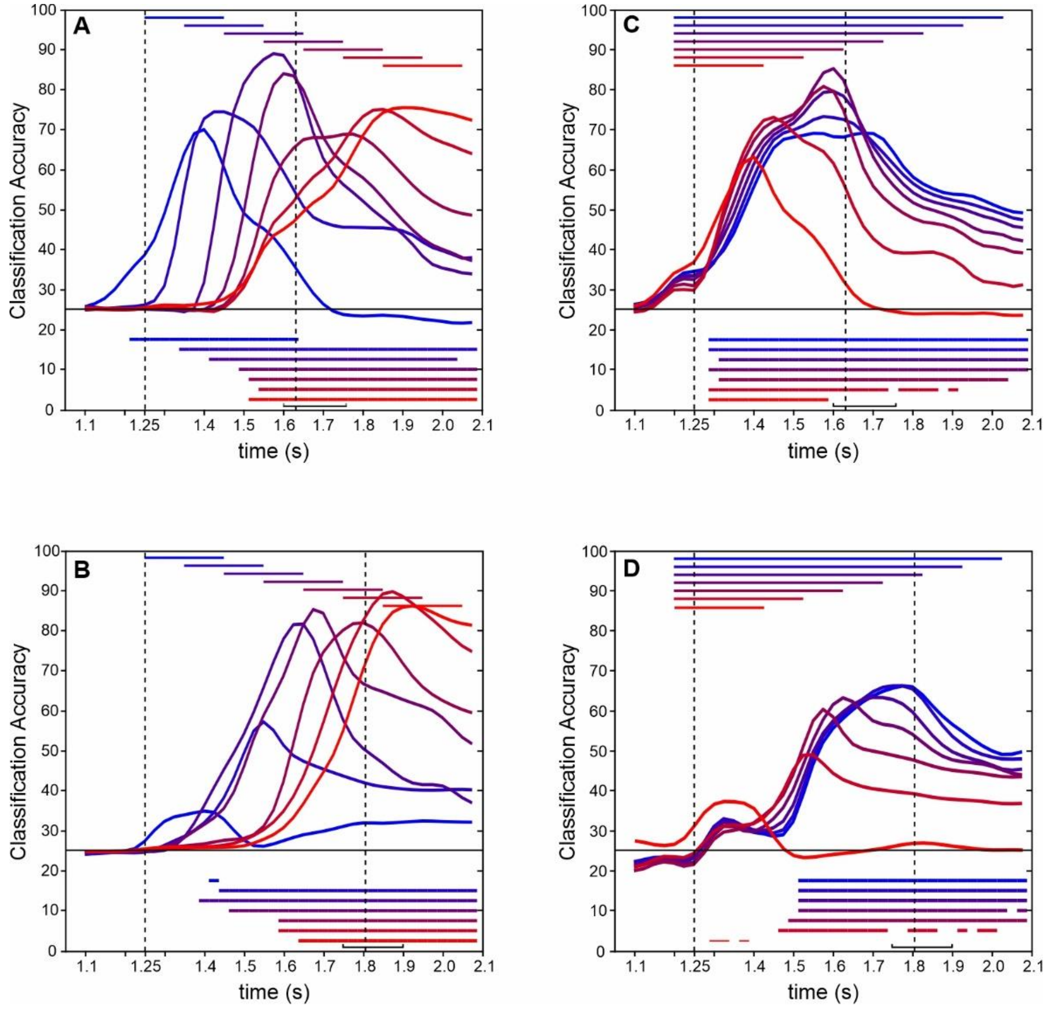

Fig. S7. Neural principal components predicted from temporally restricted kinematic windows support grasp configuration classification. Classification of grasp configuration from neural activity predicted using regression models trained on restricted kinematic windows. Classifiers were trained on measured neural data and tested on predicted neural data. **A**, **C**: MirNs-GE. **B**, **D**: MirNs-GO. Regression models were trained on 200 ms kinematic segments at different movement times (**A**, **B**) or on windows of varying duration (**C**, **D**). Shaded curves correspond to different training windows (color-coded horizontal bars above panels). Horizontal bars below plots indicate significant classification (non-parametric permutation test; 500 label shuffles; one-sided $p < 0.002$). Dashed vertical lines mark movement onset (1.25 s) and movement end.

Fig. S8.

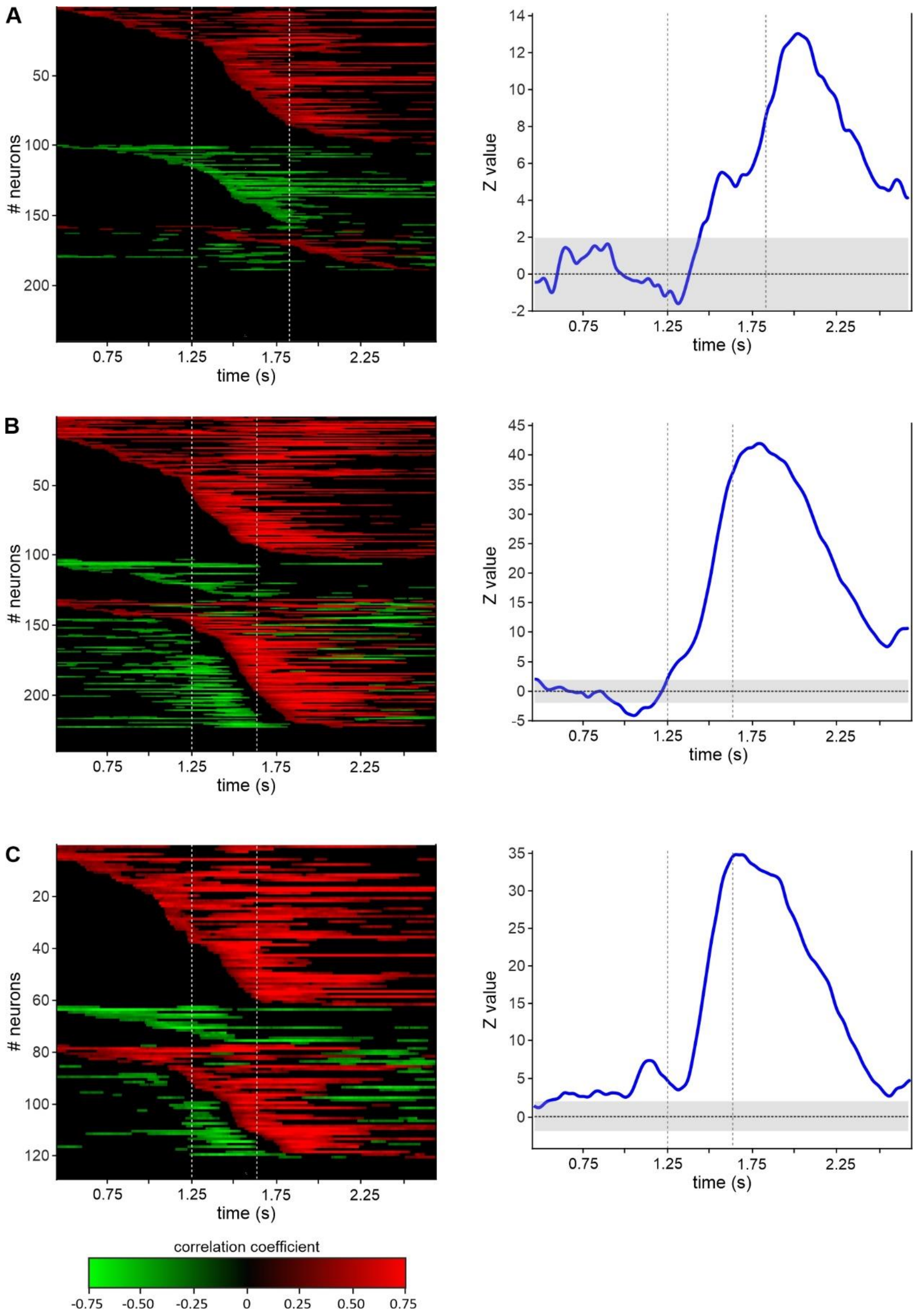
A
B
C
neurons
time (s)
Z value
0.75
1.25
1.75
2.25
correlation coefficient
-0.75
-0.50
-0.25
0
0.25
0.50
0.75

Fig. S8. Movement duration correlates with neuronal discharge. Left panels show Pearson correlation coefficients (red: positive; green: negative) between trial-by-trial movement duration and firing rate for individual neurons: MirNs during GO (**A**), MirNs during GE (**B**), and non-MirNs (**C**). Correlations are shown only at time bins meeting significance criteria (two-tailed Pearson correlation; $p < 0.05$; ≥11 consecutive bins). Right panels show Fisher z-transformed correlation coefficients averaged across neurons. Gray shading indicates population-level significance ($|z| > 1.96$; weighted z-test; $p < 0.05$).

Fig. S9.

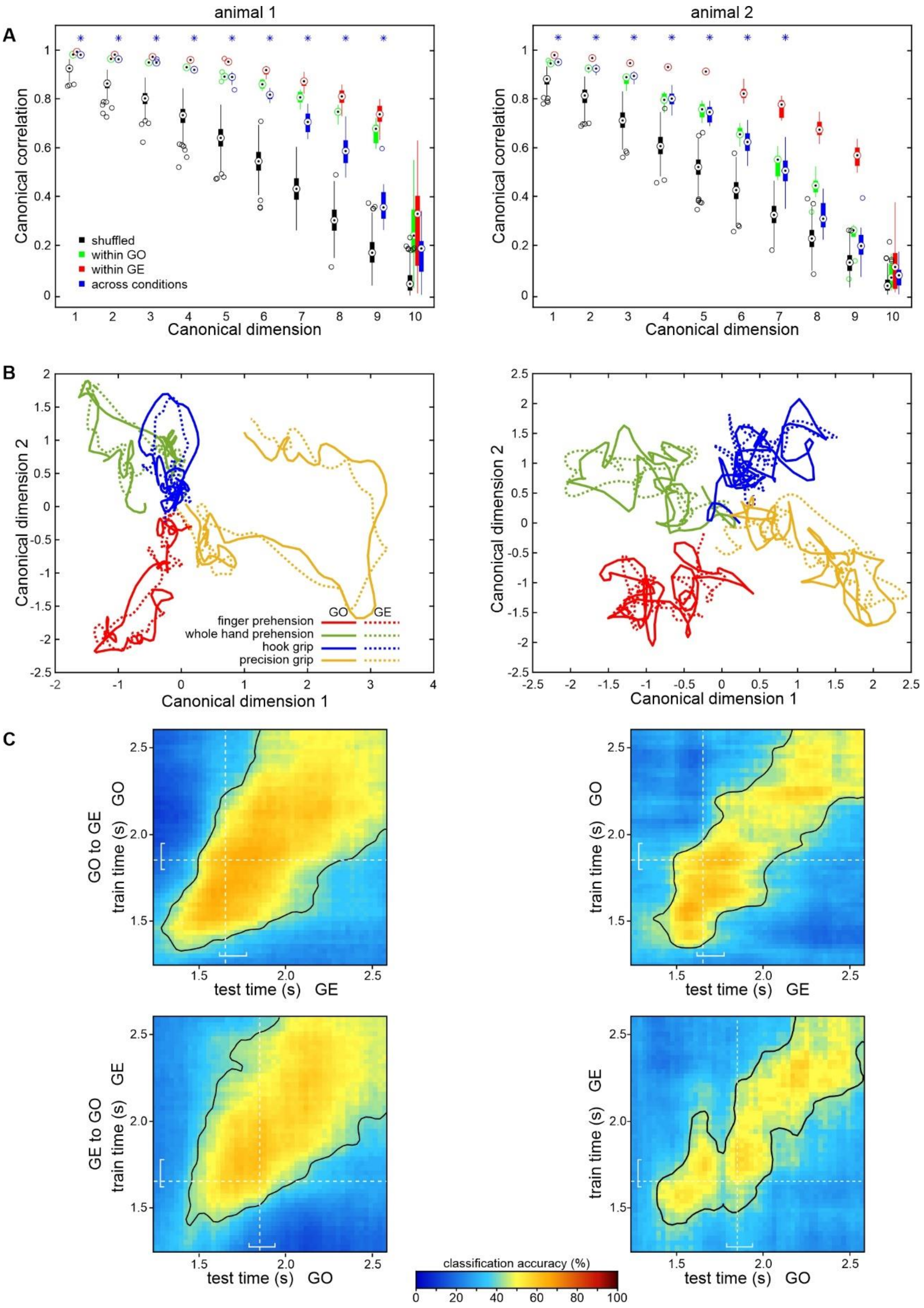
animal 1
animal 2
A
B
C
Canonical correlation
Canonical dimension
shuffled
within GO
within GE
across conditions
Canonical dimension 1
Canonical dimension 2
GO
GE
finger prehension
whole hand prehension
hook grip
precision grip
GO to GE
GE to GO
train time (s)
test time (s)
classification accuracy (%)

Fig. S9. Replication of key findings across animals. A: Cross-validated canonical correlations across the first 10 canonical dimensions within and between conditions for MirNs, shown separately for each monkey. Boxes indicate medians, interquartile ranges, whiskers denote non-outlier minima and maxima, and circles indicate outliers. Significance assessed via permutation testing (500 label shuffles; one-sided $p < 0.01$). Black denotes shuffled null distribution. **B**: Neural activity projected onto the first two canonical dimensions, showing clustering of grasp configurations. **C**: Cross-temporal grasp configuration classification across conditions (GO → GE; GE → GO). Black contours indicate significant classification (non-parametric permutation test; 500 label shuffles; one-sided $p < 0.002$). Time aligned to movement onset (1.25 s); dashed lines indicate movement end.

Fig. S10.

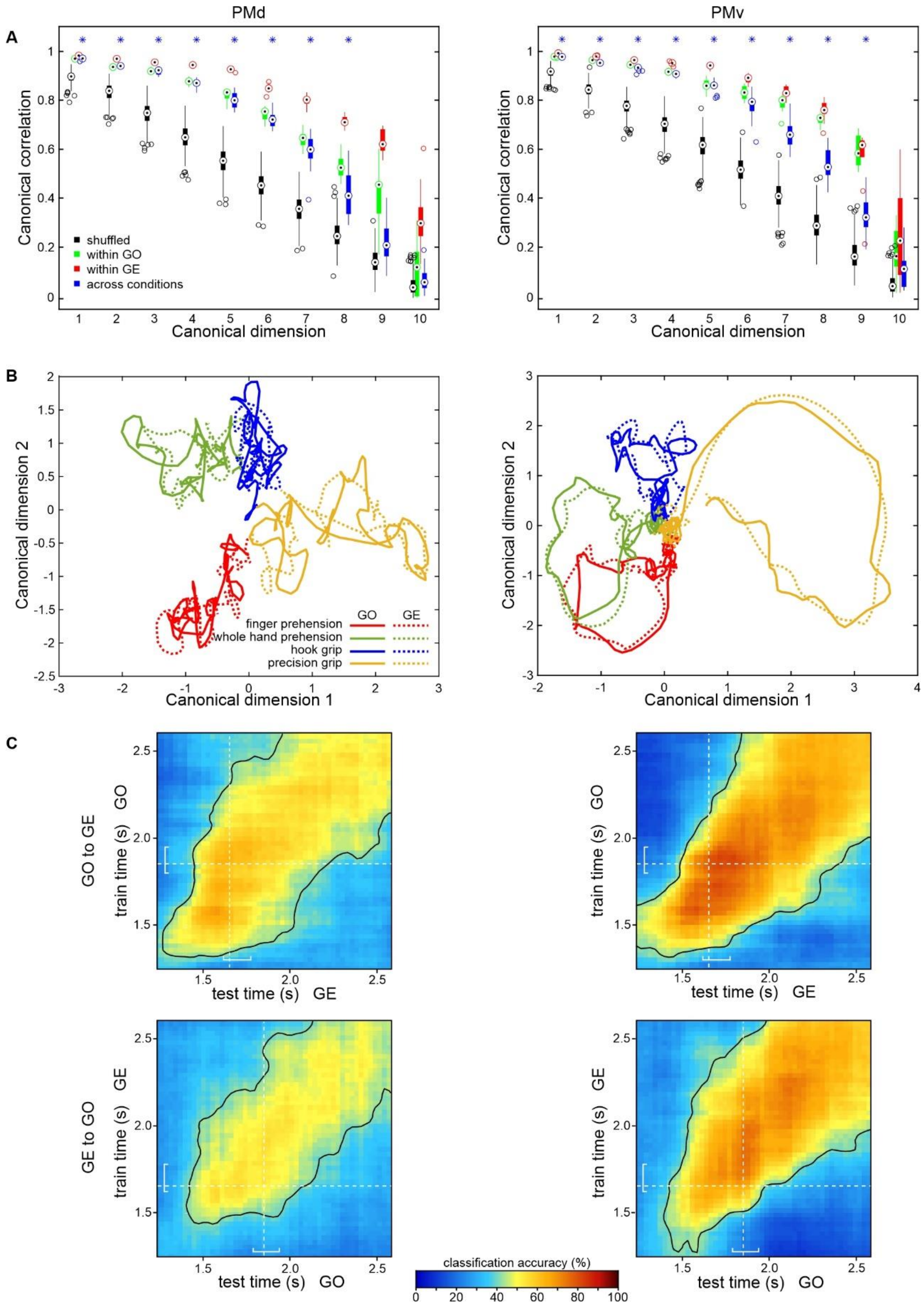
A
B
C
PMd
PMv
Canonical correlation
Canonical dimension
shuffled
within GO
within GE
across conditions
Canonical dimension 1
Canonical dimension 2
GO
GE
finger prehension
whole hand prehension
hook grip
precision grip
GO to GE
GE to GO
train time (s)
test time (s)
classification accuracy (%)

Fig. S10. Replication of key findings across cortical areas. A: Cross-validated canonical correlations across the first 10 canonical dimensions within and between conditions for MirNs, shown separately for PMd and PMv. Boxes indicate medians, interquartile ranges, whiskers denote non-outlier minima and maxima, and circles indicate outliers. Significance assessed via permutation testing (500 label shuffles; one-sided $p < 0.01$). Black denotes shuffled null distribution. **B:** Neural activity projected onto the first two canonical dimensions, illustrating clustering of grasp configurations within each area. **C:** Cross-temporal grasp configuration classification across conditions (GO → GE; GE → GO) for each area. Thin black contours indicate significant classification (non-parametric permutation test; 500 label shuffles; one-sided $p < 0.002$). Time aligned to movement onset (1.25 s); dashed lines indicate movement end.

Table S1.

	PMd			PMv			total		
both types	220 (182)	m1:	126 (106)	213 (187)	m1:	156 (137)	433 (369)	m1:	282 (243)
		m2:	94 (76)		m2:	57 (50)		m2:	151 (126)
MirNs	142 (116)	m1:	80 (68)	143 (124)	m1:	101 (89)	285 (240)	m1:	181 (157)
		m2:	62 (48)		m2:	42 (35)		m2:	104 (83)
non-MirNs	78 (66)	m1:	46 (38)	70 (63)	m1:	55 (48)	148 (129)	m1:	101 (86)
		m2:	32 (28)		m2:	15 (15)		m2:	47 (43)

Table S1. Numbers of neuron types per area and animal. Values in parentheses indicate neurons recorded in ≥9 trials per grip and condition and included in the analyses.

Table S2.

KINEMATIC PARAMETER			entire dataset		monkey 1		monkey 2		PMd		PMv		non-MirNs
			R^2		R^2		R^2		R^2		R^2		R^2
			GE	GO	GE	GO	GE	GO	GE	GO	GE	GO	GE
aperture	position		**0.533**	**0.855**	0.565	0.857	0.700	0.771	0.592	0.851	0.707	0.865	0.530
	velocity		**0.543**	**0.713**	0.661	0.634	0.582	0.679	0.713	0.713	0.667	0.640	0.805
wrist	position	X	**0.814**	**0.970**	0.970	0.973	0.904	0.925	0.952	0.962	0.962	0.971	0.984
		Y	**0.838**	**0.973**	0.885	0.971	0.853	0.962	0.749	0.970	0.845	0.979	0.810
		Z	**0.764**	**0.949**	0.914	0.957	0.913	0.950	0.948	0.913	0.899	0.946	0.956
	velocity	X	**0.418**	**0.928**	0.790	0.910	0.518	0.909	0.789	0.894	0.637	0.910	0.818
		Y	**0.586**	**0.943**	0.800	0.952	0.799	0.873	0.848	0.920	0.827	0.935	0.861
		Z	**0.448**	**0.957**	0.739	0.965	0.577	0.924	0.787	0.936	0.649	0.957	0.793
thumb	position	X	**0.741**	**0.968**	0.801	0.900	0.736	0.861	0.799	0.910	0.788	0.928	0.764
		Y	**0.456**	**0.971**	0.755	0.815	0.766	0.525	0.736	0.739	0.718	0.793	0.743
		Z	**0.738**	**0.868**	0.821	0.722	0.822	0.693	0.862	0.653	0.788	0.776	0.809
	velocity	X	**0.309**	**0.942**	0.459	0.750	0.298	0.754	0.513	0.724	0.345	0.712	0.584
		Y	**0.154**	**0.932**	0.194	0.489	0.222	0.439	0.248	0.512	0.185	0.394	0.172
		Z	**0.134**	**0.802**	0.441	0.686	0.225	0.679	0.421	0.763	0.332	0.559	0.558
index	position	X	**0.628**	**0.976**	0.764	0.881	0.749	0.872	0.843	0.868	0.770	0.906	0.833
		Y	**0.753**	**0.972**	0.770	0.809	0.695	0.686	0.773	0.733	0.721	0.852	0.554
		Z	**0.768**	**0.921**	0.841	0.658	0.814	0.720	0.874	0.503	0.843	0.724	0.869
	velocity	X	**0.359**	**0.968**	0.426	0.753	0.293	0.819	0.496	0.788	0.368	0.725	0.546
		Y	**0.129**	**0.961**	0.268	0.591	0.170	0.600	0.281	0.728	0.222	0.509	0.399
		Z	**0.208**	**0.766**	0.482	0.665	0.255	0.649	0.474	0.742	0.327	0.504	0.609

Table S2. Regression R^2 values for predicting action kinematics from neural activity during GE and GO.

Table S3.

PCs	entire dataset		monkey 1		monkey 2		PMd		PMv		non-MirNs
	R^2		R^2		R^2		R^2		R^2		R^2
	GE	GO	GE	GO	GE	GO	GE	GO	GE	GO	GE
**1**	**0.848**	**0.945**	0.840	0.947	0.829	0.936	0.869	0.933	0.513	0.933	0.918
**2**	**0.502**	**0.926**	0.480	0.867	0.500	0.928	0.704	0.941	0.776	0.923	0.865
**3**	**0.534**	**0.711**	0.457	0.455	0.538	0.738	0.359	0.385	0.478	0.239	0.380
**4**	**0.193**	**0.330**	0.270	0.678	0.219	0.121	0.171	0.551	0.247	0.734	0.111
**5**	**0.357**	**0.588**	0.377	0.446	0.289	0.393	0.540	0.658	0.211	0.405	0.122
**6**	**0.104**	**0.316**	0.086	0.393	0.486	0.263	0.484	0.144	0.157	0.451	0.416
**7**	**0.684**	**0.238**	0.644	0.189	0.043	0.113	0.115	0.172	0.519	0.203	0.360

Table S3. Regression R^2 values for predicting neural principal components from monkey and human kinematics.
